# Aerosol capture and coronavirus spike protein deactivation by enzyme functionalized antiviral membranes

**DOI:** 10.1038/s43246-022-00256-0

**Published:** 2022-05-24

**Authors:** Rollie Mills, Ronald J. Vogler, Matthew Bernard, Jacob Concolino, Louis B. Hersh, Yinan Wei, Jeffrey Todd Hastings, Thomas Dziubla, Kevin C. Baldridge, Dibakar Bhattacharyya

**Affiliations:** 1 Department of Chemical and Materials Engineering, University of Kentucky, Lexington, KY 40506, USA.; 2 Department of Molecular and Cellular Biochemistry, University of Kentucky, Lexington, KY 40506, USA.; 3 Department of Chemistry, University of Kentucky, Lexington, KY 40506, USA.; 4 Department of Electrical and Computer Engineering, University of Kentucky, Lexington, KY 40506, USA.; 5 These authors contributed equally: Ronald J. Vogler, Matthew Bernard.

## Abstract

The airborne nature of coronavirus transmission makes it critical to develop new barrier technologies that can simultaneously reduce aerosol and viral spread. Here, we report nanostructured membranes with tunable thickness and porosity for filtering coronavirus-sized aerosols, combined with antiviral enzyme functionalization that can denature spike glycoproteins of the SARS-CoV-2 virus in low-hydration environments. Thin, asymmetric membranes with subtilisin enzyme and methacrylic functionalization show more than 98.90% filtration efficiency for 100-nm unfunctionalized and protein-functionalized polystyrene latex aerosol particles. Unfunctionalized membranes provided a protection factor of 540 ± 380 for coronavirus-sized particle, above the Occupational Safety and Health Administration’s standard of 10 for N95 masks. SARS-CoV-2 spike glycoprotein on the surface of coronavirus-sized particles was denatured in 30 s by subtilisin enzyme-functionalized membranes with 0.02-0.2% water content on the membrane surface.

The use of respiratory face masks provides significant reduction of coronavirus spread, as viral spread has been proven to occur primarily via two modes of transmission: droplet spread (direct) and inhalation of infectious airborne aerosols (indirect)^1-3^. The efficacy of filtering facepiece respirators (FFRs), such as N95 masks, has been extensively investigated in recent years, due to the severe acute respiratory syndrome coronavirus 2 (SARS-CoV-2) pandemic and influenza. These National Institute for Occupational Health and Safety (NIOSH)-approved devices consist of three to four hydrophobic layers of non-woven polypropylene (PP) fibers with an electrically-charged filtration layer for improved particle capture and rejection^4^. The outer layers typically consist of spun-bound (SB) fibers with a relatively large surface area (~2 m^2^/g), while the inner layer consists of melt-blown (MB) fibers of smaller surface area (~0.2 m^2^/g) with dipole charges^5,6^. N95 masks are the most commonly-used mask in the healthcare industry, yet do not offer consistent virus transmission prevention. Due to the open structure of the fibrous layers, such masks are limited in the capture of smaller-sized particles (<300 nm), and in the ability to maintain electrically-charged properties (lost during decontamination processes)^5,6^. Lee et al. found that only 70% of 45 tested N95 masks (4 different models) offered >90% rejection of sodium chloride (NaCl) aerosolized particles of coronavirus-like size (40–200 nm)^6^, indicating significant danger for virus transmission. Therefore, the innovative development of smart filtration materials with low airflow resistance that can filter, capture, and deactivate aerosolized virus particles can provide immense human health and industrial work-place benefits.

Water-filtration membranes have been utilized for virus filtration, but their applications to aerosol capture have not been well-studied and could enable promising new developments for respiratory face masks and enclosed space-environmental filters. Thin-film polymeric water-filtration membranes, such as poly(vinylidene fluoride) (PVDF), could mitigate this danger via air-filtration and offer greater aerosolized particle capture than N95 masks, due to precise control of the membrane pore size and structure^7^. PVDF has been shown to be effective in capturing coronavirus-simulated aerosols^8,9^. PVDF membranes with specific variables (thickness, porosity, pore size) could be developed to optimize air permeability and pressure drop while allowing for high capture and filtration efficiency of coronavirus-size particles. Furthermore, to avoid direct contact with the individual and to enable easy replacement, the membrane material could work as a mask insert between two other layers. Overall, to develop such membranes, a structural understanding of how membrane characteristics affect the air permeability and aerosolized particle capture must be achieved. Several fundamental equations that correlate membrane variables to flow, in addition to air nano-flow transport derivations, can be utilized to determine such relationships.

Further protection with air filtration materials has been achieved by functionalizing the material with an antiviral agent for reducing/eliminating coronavirus infectivity. This has been accomplished with different materials, such as silver nanoparticles^10^, copper sulfide^11^, salts^12^, and active herbal ingredients from plant species^13^, yet significant limitations exist, such as time for infectivity elimination (~30 min) or potential toxic/irritant nature of added material (heavy metals and salts). Similar infectivity deactivation could be achieved quickly (<60 s) without the addition of toxic material via enzyme functionalization of membrane surface and pores. Studies have indicated that certain enzymes can denature proteins by causing a small conformation change^14^, such as a minute change in the dihedral angles of amino acid residues^15,16^. In general, protein denaturation results in the loss of the native protein configuration and functionality, which often exposes protected hydrophobic regions of the protein, and can occur via heat, urea, pH, and alcohol treatment^17^. Coronaviruses contain several primary structural proteins, including the small envelope glycoprotein, the nucleocapsid protein, the spike glycoprotein and the membrane glycoprotein^18^. The spike glycoprotein (SGP, S-Protein), with a molecular weight of ~150 kDa for SARS-CoV-2, has become the focus of coronavirus research, as it facilities the infection of host cells through binding to specific host surface receptors^19-22^. It could be hypothesized that disrupting/denaturing the SGP would deactivate the virus. Overall, the enzyme functionalization process of membranes is well-established^23-26^ and could allow for inactivation of coronavirus droplets that reach the membrane.

A stable, serine protease enzyme, such as subtilisin Carlsberg, could be ideal for protein denaturation in filter applications, as the enzyme is stable in a broad range of conditions (even in anhydrous solvent with minimal water presence), the enzyme’s functionality is well understood, and substrate-binding modifications are highly studied^27-29^. Enzyme functionalization of the membrane could be further enhanced by adding poly(methacrylic acid) (PMAA), a non-toxic polymer that can electrostatically interact with an enzyme with a high isoelectric point (pI) value, such as subtilisin^30-32^. The presence of water has been proven to be necessary for enzyme structure and function, thus functionalization with PMAA, which can retain water^33^, could enhance enzyme longevity as well. Furthermore, such material for membrane functionalization presents low toxicity upon human exposure. Subtilisin is a common enzyme utilized in commercial laundry detergent^34^ and exposure to mammalian animals has shown no acute or subchronic adverse effects^35^. PMAA is considered a highly biocompatible material^36^ with low toxicity that is commonly used for in vivo and in vitro applications^37^.

This paper investigated the development of a personal and closed-environment membrane filter with enhanced aerosol and particle capture, along with the ability to inactivate coronaviruses (specifically, SARS-CoV-2) through enzyme functionalization, greatly reducing both individual transmissibility of the virus and overall disease spread. Specific membrane variables appropriate for mask and air-filtration applications were developed and compared to commercial respiratory masks. The immobilization of subtilisin onto PVDF400 and PMAA-functionalized PVDF400 (PMAA-PVDF) surfaces was investigated using batch and convective flow functionalization methods. The membrane’s enhanced particle filtration and capture as a function of particle size were tested and compared to commercial N95 masks. The denaturation of the SGP of SARS-CoV-2 (wild type) by enzyme-functionalized membranes was tested under low hydration conditions.

## Results

### Membrane characterization.

Water-filtration membranes are used for many purposes, ranging from suspended solid separation (microfiltration-MF) and colloidal macromolecule filtration (ultrafiltration-UF) to the separation of low molecular weight molecules and monovalent salts (nanofiltration-NF)^38,39^. These membranes are made out of different materials, such has polymers, carbon nanotubes, or graphene oxide^40,41^, and have different filtration uses, due to their unique flow profiles, transfer mechanisms, and pore size range. Each flow profile is controlled by its respective membrane properties (porosity, pore diameter, thickness), which were experimentally quantified for the commercial membranes hydrophilized PVDF400-MF (porosity: 0.46, thickness: 165 μm, average pore diameter: 45 nm with ~5% of pores with diameter 100–120 nm), polysulfone PS35-UF (porosity: 0.03, thickness: 224 μm, average pore diameter: 15–20 nm), and NF270-NF (porosity: NA, thickness: 152 μm, average pore diameter: 0.08 nm) to better understand the fluid/airflow through these membranes.

**Fig. 1:**
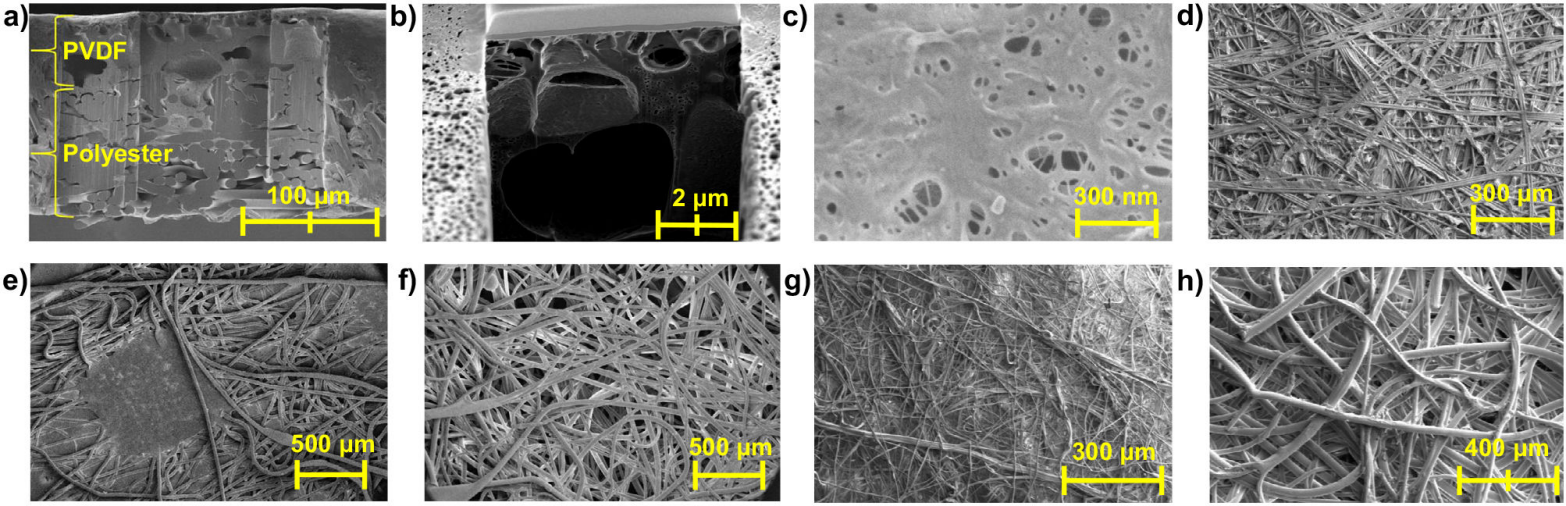
SEM of PVDF400 commercial membrane and N95 mask. **a** full thickness cross-section of PVDF400 and **b** PVDF-only layer cross-section. Surface of **c** PVDF layer and **d** polyester support layer. SEM images of N95 layers (**e**) 1, (**f**) 2, (**g**) 3, and (**h**) 4. In mask orientation, layer 1 is exposed to the open environment and layer 4 is exposed to the inside of the mask. *Layer 3 is referred to as the “separating” layer, as it had the highest flow resistance*.

Many water-filtration membranes are composed of two layers: a thin selective layer that is utilized for separation/filtration purposes and a thicker open support structure. This design reduces the applied pressure needed for flow (lower energy requirement and operating cost) and the probability of pore structure disruption during high-pressure filtration. PVDF400 membranes follow this structure and are composed of a PVDF separating layer (thickness~60 μm, average pore size~45 nm), which is the primary filtration/separation layer for water-based and aerosolized particles, and a polyester support layer for enhanced mechanical strength (thickness~100 μm), which was confirmed with scanning electron microscopy (SEM) in Fig. 1a-d. Pore diameter distribution was confirmed using ImageJ software (Supplementary Fig. 1).

Contact-angle is an important measure of membrane fouling rate and a common test for mask materials^42^. The contact-angle of unfunctionalized PVDF400 was relatively high, starting at 86.2° ± 0.5° (*t* = 2 s) and remaining over 80°, indicating a relatively-hydrophobic behavior (Supplementary Fig. 2). Upon PMAA and subtilisin functionalization, the membrane initially exhibited a high contact-angle (72.2° ± 3.2° and 53.4° ± 4.2°, respectively), which reduced to under 42° and 17°, respectively, after 38 s of drop deposition, indicating that PMAA and subtilisin functionalization introduces hydrophilic behavior into the membrane system, which is likely to further reduce membrane fouling.

### Effect of membrane characteristics on fluid flow rate.

Air and aerosol flow through respiratory face masks have been extensively studied to understand particle filtration efficiency and pressure drop across the material^43,44^. N95 and surgical masks are most commonly fabricated from multi-layer non-woven PP^45^, shown in Fig. 1e-h (Supplementary Fig. 3a for N95 layers and thicknesses, Supplementary Fig. 3b for schematic of mask layers and structures), and, due to the random nature of this system, the effects of certain mask variables on flow are more challenging to predict. Kumar and Lee reported that the complex flow of air through these mask materials can be characterized by a complex mass transfer equation, derived from the system’s momentum balances^46^.

Fluid flow through porous microfiltration or ultrafiltration membranes can be expressed in simpler terms than existing respiratory face masks, due to sieving transport flow occurring through such pores. Due to the precise structure, the effect of membrane variables on flow can be predicted more accurately than that of mask materials. Pressure-driven convective flow of an incompressible fluid through porous membranes can be expressed by the Hagan Poiseuille equation:

(1) $$J_{solvent}=\left(\begin{array}{c} membrane\\ properties \end{array}\right)\left(\begin{array}{c} fluid∕solvent\\ properties \end{array}\right)\left(\begin{array}{c} driving\\ force \end{array}\right)\;\;=\left(\frac{\varepsilon r_{p}^{2}}{8\delta \tau }\right)\left(\frac{1}{\mu }\right)(\Delta P)$$

where *J*_*solvent*_ is volumetric flux (m^3^ m^−2^ s^−1^), *ε* is membrane porosity, *μ* is dynamic liquid viscosity (kg m^−1^ s^−1^), *r_p_* is average pore radius (m), *ΔP* is pressure difference across the membrane (Pa), and *δ* is membrane thickness (m)^47^.

Airflow through porous membranes cannot be expressed using the Hagan Poiseuille equation, as it does not calculate flow as a function of density. The density of air (compressible fluid) is affected by temperature, pressure, and relative humidity^48^, which can vary in pressure-drive flow systems. Taking density into account, modeling mass airflow rate through a specific micropore can be more accurately determined using the following equation that was modified for low-pressure flow through circular pores^49^:

(2) $$\dot{m}=\left(\begin{array}{c} membrane\\ properties \end{array}\right)\left(\begin{array}{c} fluid\\ properties \end{array}\right)\left(\begin{array}{c} driving\\ force \end{array}\right)=\left(\frac{4r_{p}^{4}}{3\delta }\right)\left(\frac{\overset{‒}{P}}{RT\mu }\right)(\Delta P)$$

Where $\dot{m}$ is the air mass flow rate through a single pore (or a channel), *T* is temperature of the air (Kelvin), $\overset{‒}{P}$ is average pressure channel/channel (Pa), and *R* is the gas constant. *Kn* is included in the original equation (proven to be negligible), which is calculated by the ratio of the mean free path of the particle (*λ*) and the length of the flow channel (L) (Kn = *λ*/L). Density corrections are made in the fluid properties, as the ideal gas assumption is utilized, resulting in 1/ρ (density) from average pressure over R×T. From Eqs. 1 & 2, a positive relationship between flux and porosity, and flux and pore radius, as well as an inverse relationship between flux and thickness, can be established.

**Fig. 2:**
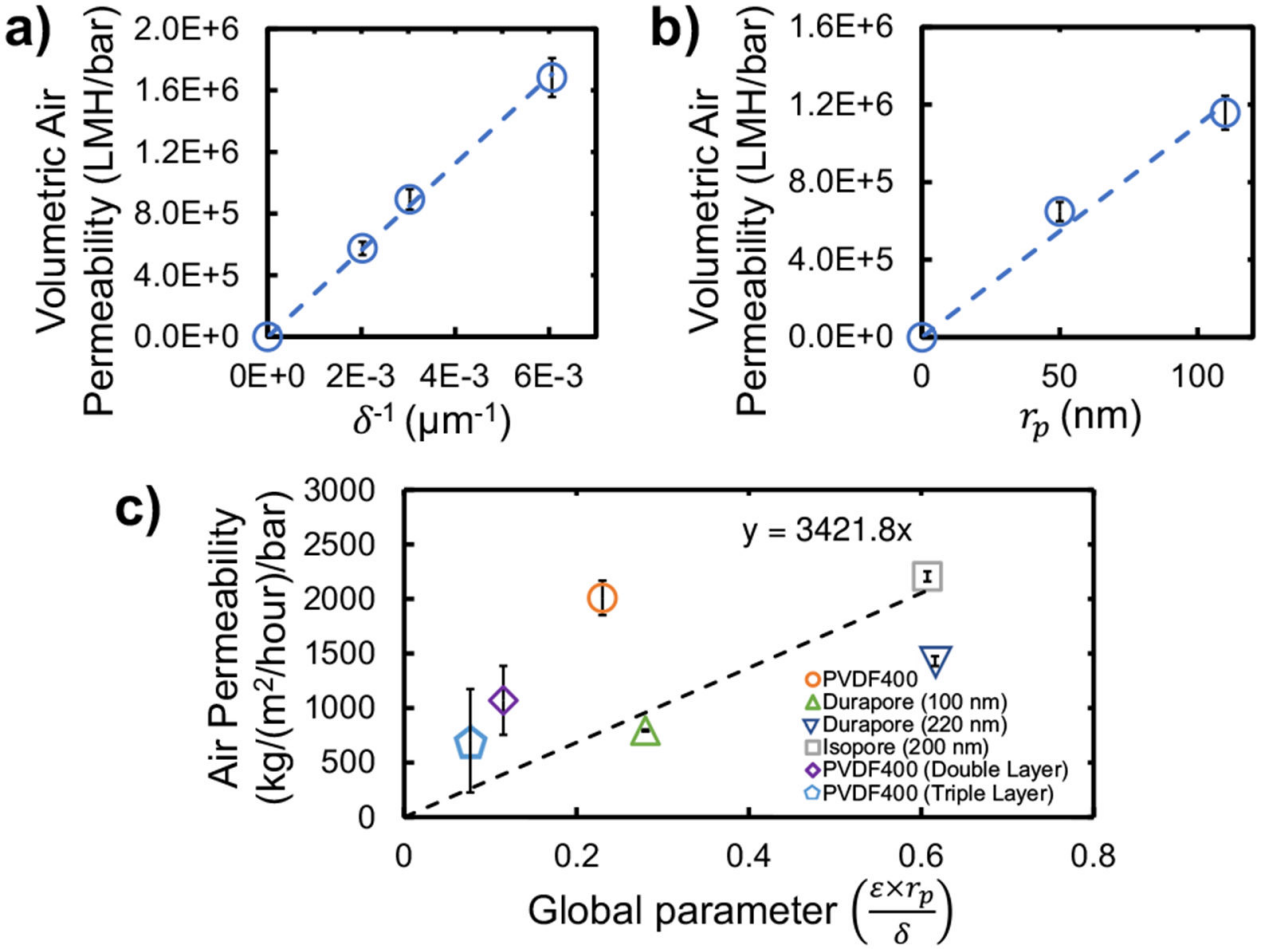
Membrane air permeability relationship to key membrane parameters. The experimental relationship between dry-air permeability (LMH or liters per m^2^ per hour for volumetric, kg per m^2^ per hour for mass) and **a** the inverse of membrane thickness via PVDF400 stacking with no spacing in between membranes to increase thickness (blue circles with blue dashed line) and **b** pore radius (via Durapore membranes with varying pore size) for water-filtration membranes (blue circles with blue dashed line). **c** Experimentally-determined linear relationship between air permeability and a global parameter for membrane variables (black dashed line). All membranes used displayed hydrophilic behavior (orange circle for PVDF400, green triangle for Durapore-100 nm, dark blue invert pyramid for Durapore-220 nm, gray square for Isopore-200 nm, purple diamond for PVDF400-Double Layer and light blue pentagon for PVDF400-Triple Layer). PVDF400 flows are at normal orientation. Data was collected via dry-air-filtration (0% relative humidity) using in-line flow cell with an area of 9.23 cm^2^. Measured flow rate measurements normalized at STP. Error bars represent the standard deviation of 3 different measurements taken on the samples.

For membrane-based air-filters to be viable, their breathability must be comparable to that of commercial face masks. To reach this benchmark, membrane variables (membrane properties of Eqs. 1 and 2) can be adjusted to obtain predictable flux changes^50^. To test the permeability control by membrane variables, the relationship between membrane permeability and certain variables (thickness, pore radius) were tested experimentally for commercial water-filtration membranes. The thickness relationship was tested by PVDF400 stacking (constant porosity and pore size) in Fig. 2a, while the relationship of pore size was tested by testing two commercial PVDF Durapore membranes of different pore size (constant porosity and thickness) in Fig. 2b. The resulting trendlines for inverse thickness and pore radius indicate a semi-linear relationship between these variables and air permeability, thus confirming the ability to precisely control air permeability and pressure drop of water-filtration membranes.

MF membranes are often characterized by their water or air permeability, but there have not been significant studies on modeling the air permeability of porous commercial water-filtration membranes. The relationship between flow and membrane variables can be utilized to predict the airflow through MF membranes. Membrane tortuosity will affect the air permeability, but is not considered, due to the complexity of measuring it^51^. The air permeability of various MF membranes was experimentally determined and normalized with respect to porosity, pore radius, and thickness (relationship from Fig. 2a, b) to estimate the air permeability of polycarbonate Isopore membranes (properties in Supplementary Table 1) (Fig. 2c). Based on the relationship between air permeability (kg/m^2^/h) and a global parameter for membrane variables, Isopore membranes were estimated to have an air permeability of 2077 kg/m^2^/h. This value was corroborated by experiments, which measured an Isopore permeability of 2206 ± 45 kg/m^2^/h, demonstrating that the air permeability of MF water-filtration membranes can be reliably estimated using membrane variables.

### Performance comparison between membranes and commercial mask performance.

**Fig. 3:**
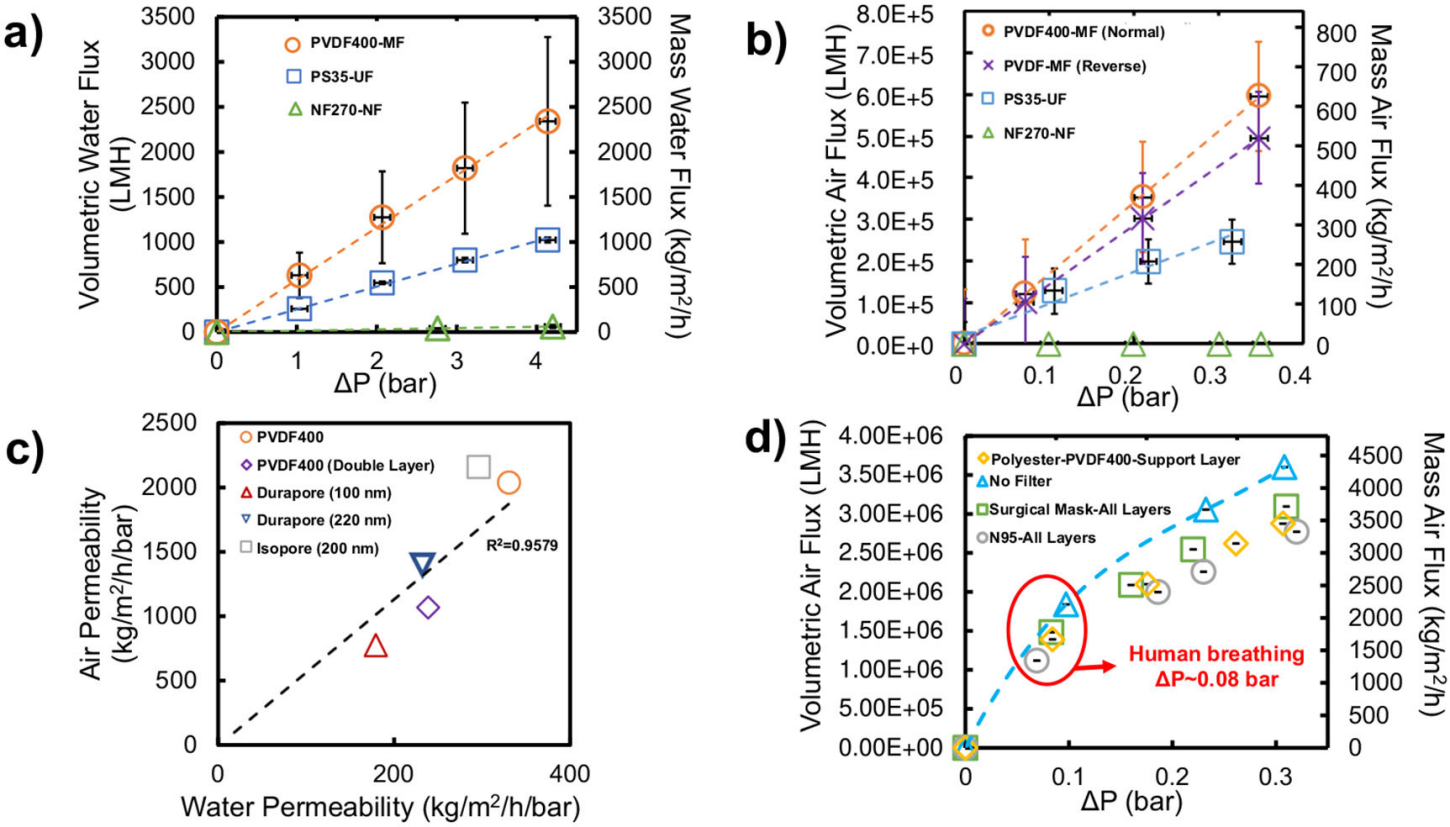
Characterizing flux through various types of membranes and air filter material. **a** Water and **b** dry-air (0% RH) flux results for PVDF400-MF (orange circle and dashed line for normal orientation, purple cross mark and dashed line for reverse), PS35-UF (blue square and dashed line), and NF270-NF (green triangle and dashed line) membranes as a function of pressure drop (ΔP) (LMH or liters per m^2^ per hour for volumetric, kg per m^2^ per hour for mass). **c** Relationship between water permeability and air permeability for specific single sample MF membranes (orange circle for PVDF400, gray square for isopore-200 nm, dark blue invert pyramid for Durapore-220 nm, purple diamond for PVDF400-Double Layer, red triangle for Durapore-100 nm, and black dashed line for linear trendline). Note Durapore (100 nm) is hydrophilic. **d** Comparison of mask material (all layers) with PVDF400 polyester backing material (yellow diamond for polyester backing, green square for surgical mask-all layers, and gray circle for N95-all layers). No filter flow is airflow through the filtration cell with no filter/mask/membrane insert (light blue triangle and dashed line), indicating maximum flow through the cell at that applied pressure. Water flux results were obtained using a dead-end cell with a membrane are of 13.2 cm^2^. Air flux data were collected using in-line flow cell with an area of 9.23 cm^2^. Error bars represent standard deviations of triplicate data. Both horizontal axis (left and right) apply for all data displayed. Flow rate measurements normalized at STP. The PVDF400 membrane was tested in two orientations for air-filtration: with the surface facing the feed side (Normal) and the surface facing the permeate side (Reverse). Error bars represent the standard deviation of 3 different measurements taken on the samples.

Commercial water-filtration membranes have been extensively studied for liquid-phase virus particle separation/capture^52^, but their efficiency for air-phase aerosol separation/capture is less commonly investigated^53^. The dry-air flux of PVDF400 (MF), PS35 (UF), and NF270 (NF) membranes were experimentally determined and compared to their corresponding water fluxes in Fig. 3a, b. Similar trends between dry-air and water flux were observed, such as a linear correlation between applied pressure and air flux and the MF membrane having the highest permeability, thus indicating that these water-filtration membranes could be appropriate for airflow and aerosol capture applications. Additionally, the effect of air relative humidity (RH) on air permeability of both unfunctionalized and PMAA-functionalized PVDF membranes was shown to be negligible (within one standard deviation) (Supplementary Fig. 4).

A relationship was developed between the water and air permeability of water microfiltration membranes to prove that the air permeability of microfiltration membranes can be estimated using their water permeability without the need for membrane variable values (Fig. 3c). Water flow (up to 4 bar) and airflow (up to 0.35 bar) were calculated to be laminar for these membranes (based on Reynold’s Number). Porosity and pore size of PS35, Durapore (100 nm and 220 nm), and Isopore membranes were confirmed using SEM (Supplementary Fig. 5). NF270 surface imaging using SEM is limited, as this membrane is a nonporous dense membrane with diffusion through intermolecular spacing (stated as pore size) as the primary means of flux through the membrane. PVDF400 triple layer water permeability was not obtained due to material thickness limitations of the filtration cell.

PVDF400 MF membranes were determined to be best for filter usage (compared to UF and NF membranes) because of their superior air permeability without having an average pore size greater than 100 nm and because PVDF membranes have been proven to have high aerosol filtration capabilities upon proper design^8^. Furthermore, PVDF400 is a promising option, due to its tortuous pores, which has been proven by the Centers for Disease Control and Prevention (CDC) to increase the chance of particle capture, as opposed to a non-tortuous Isopore membrane^54^. The dry-air permeability of PVDF400’s polyester support layer was experimentally compared to surgical and N95 masks (Fig. 3d). The air permeability of the N95 and surgical mask are superior to that of the commercial PVDF400 membrane, indicating that a PVDF membrane with different properties would be necessary to match the flow capabilities of commercial masks. The airflow through the N95 mask, surgical mask, and polyester support were found to be laminar (based on Reynold’s Number calculations).

PVDF membranes can be fabricated specifically for high gas permeabilities^8^, indicating that PVDF400 performance is not the upper limitation of this material’s permeability. Using previously-determined relationships (Figs. 2 & 3) and membrane variables from existing commercial membranes (Supplementary Table 1), computational modeling was conducted to determine properties of a PVDF membrane that can match the air flux of commercial N95 masks at the average human breathing pressure of 0.08 bar^55^. Two membrane designs with specific membrane variables are proposed (Supplementary Table 2). The airflow rates of these proposed membranes indicate that PVDF membranes can be fabricated to have comparable air permeability to that of respiratory face masks at human breathing pressures. Furthermore, pressure drop is often utilized as a measure of breathing resistance or “breathability” for mask material and was measured for all membrane and mask materials at a dry air flow rate of 7.5 L/min (breathing flow rate) (Supplementary Fig. 6). The breathability of such filter material must be high for masks and low-energy enclosed-environment filters. The pressure drop of the unfunctionalized and functionalized PVDF400 membranes were found to be 5–10 times higher than that of N95 masks, indicating that a PVDF membrane with different membrane properties would have to be fabricated for commercial use. Using similar relationships and membrane variables, the pressure drop of the hypothetical model membranes 1 and 2 were determined to be roughly 0.067 ± 0.027 and 0.040 ± 0.016 bar, respectively, at breathing flowrate, showing that PVDF membranes for functionalization could be fabricated with equal or higher breathability to N95 masks.

Aerosolized particles are the primary means of transport for coronaviruses and can be captured by materials via four main mechanisms: inertial impaction, direction interception, diffusional interception, and electrostatic effects^56,57^. Additionally, filters have three main modes of particle droplet filter interaction: stick (particle sticks to material), splash/rebound (particles “bounces off” material), and penetration (particle penetrates through material)^58^. The mechanism that occurs can vary greatly, as it is affected by particle size, the material, and, for membranes, which pore the particle is entering through. For respiratory face masks, aerosol filtration and capture are crucial for the protection of the wearer and has been widely studied in recent years^44,59,60^. PVDF400 membranes can exhibit a dual capture mode, depending on the orientation used. Aerosol filtration differences with varying orientation are most likely attributed to the method of particle capture, where with normal orientation, particles are captured on the PVDF surface and form a cake layer of particles (Supplementary Fig. 7a), while, with reverse orientation, particles penetrate and then are captured inside the polyester layer pores (Supplementary Fig. 7b), which offers a flexible filtration material ideal for mask and enclosed-environment filtration applications, respectively per orientation.

In this study, PVDF400 membranes were functionalized with PMAA to increase the hydration ability of membrane and increase the enzyme functionalization capacity of the membrane, without significantly reducing air permeability or increasing material biohazardous nature via surface and pore fouling, a common occurrence in highly used masks. Prior to enzyme testing, polystyrene latex (PSL) particles with sizes similar to the SARS-CoV-2 virus (diameter~100 nm) were utilized to test aerosol filtration efficiency of the membranes. The PSL concentrations tested (~37,000 aerosol particles/L in 0.3–0.5 μm range, indicating aggregation) were significantly higher than realistic viral aerosol concentrations found in literature^3,61^ to investigate successful membrane application in severe situations, emphasize size-exclusion differences, and highlight long-term material fouling. PSL particles were not neutralized before filtration efficiency tests were conducted, which is a current limitation of this study. However, all filtration tests were performed under comparable conditions and should still be viable for comparison within this work. Literature indicates that there are minimal differences in filtration efficiency of N95 masks for neutralized versus non-neutralized particles in the size ranges measured here^62^. Future work can be conducted on this topic.

**Fig. 4:**
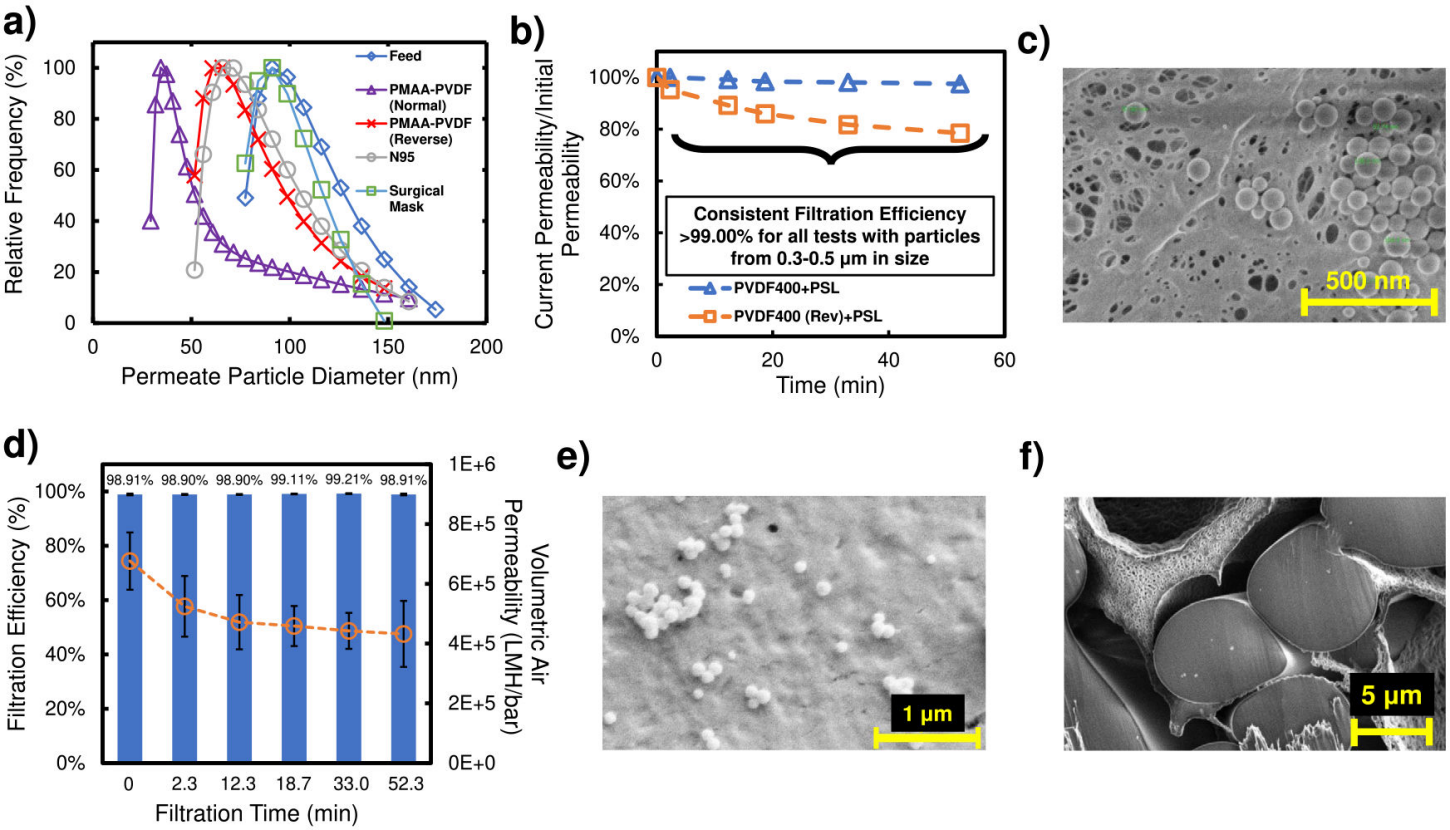
PSL aerosol filtration through membrane material. **a** PSL particle size distribution (22.7 °C) at an atomizer inlet pressure of 0.7 bar and a relative humidity (RH) of 68% after 20 min of filtration. Particle size quantified using DLS (volume-weighted measurements). N95 and surgical masks consists of all layers (purple triangle and line for PMAA-PVDF-Normal, red cross mark and line for PMAA-PVDF-Reverse, gray circle and line for N95, blue diamond for feed, and green square for surgical mask). **b** PSL particle air-filtration and permeability drop of unfunctionalized PVDF400 in normal (blue diamond and dashed line) and reverse orientation (orange square and dashed line). This result is from a single sample with <0.5% deviation in triplicate measurements. Feed air concentration was ~37,000 0.3–0.5 μm aerosol particles/L with RH of 21%. Particle count measured using Met One Instruments’ GT-526S particle counter. Flow rate measurements normalized at STP. **c** SEM image of a PVDF400 membrane surface after aerosol filtration (consisting of clusters of 100-nm PSL particles) for 10 min (RH = 68%). **d** PSL particle air-filtration and permeability drop of Subtilisin-PMAA-PVDF (blue bar for filtration efficiency, orange circle, and dashed line for permeability). Feed air concentration ~42,000 0.3–0.5 μm aerosol particles/L with relative humidity of 23%. Particle count measured using Met One Instruments’ GT-526S particle counter. Flow rate measurements normalized at STP. **e** SEM image of Subtilisin-PMAA-PVDF membrane surface after 100-nm PSL aerosol filtration for 52 min. **f** SEM cross-section image of polyester layer of Subtilisin-PMAA-PVDF membrane after 52 min of aerosol filtration (consisting of clusters of 100-nm PSL particles). Cross-section obtained using ion milling. Error bars represent the standard deviation of 3 different measurements taken on the samples.

The size distribution of aerosolized PSL particles that permeated PMAA-PVDF membranes were experimentally determined and compared to N95 and surgical masks (Fig. 4a). PMAA-PVDF membranes (normal orientation) displayed a significantly lower permeate particle size distribution than that of both commercial masks, while the same membrane in reverse orientation was observed to have a similar permeate particle size distribution as that of N95 masks. The commercial surgical mask displayed a permeate particle size distribution similar to that of the feed, confirming that surgical masks offer little to no filtration from coronavirus-sized particles^60,63^. Overall, this indicated that PMAA-PVDF membranes could offer greater protection to individuals from coronavirus particles than N95 and surgical masks, due to its more selective particle size filtration.

To further test the dual mode particle capture of PVDF400, the filtration of PSL particles through unfunctionalized PVDF400 was tested at normal and reverse orientation (Fig. 4b). Despite having a consistently high filtration efficiency (>99.0%) for both modes over 52 min of filtration, the reverse orientation showed a significant permeability drop, indicating that the membrane pores were being primarily clogging with captured particles and reducing airflow through the system, as opposed to primarily surface capture in normal orientation (Fig. 4c). After subtilisin (batch) and PMAA functionalization, the aerosol capture and permeability of the PVDF400 membrane was investigated (Fig. 4d). After maintaining >98.90% PSL filtration efficiency over 52 minutes, the membrane permeability dropped noticeably. This is due to particles blocking the membrane surface pores, which are smaller and fewer in number after enzyme functionalization (Fig. 4e). Despite this fouling, minimal particle capture inside the membrane was observed (Fig. 4f), thus enzyme functionalization does not change particle capture behavior of the dual mode system. Hydrodynamic diameter of PSL particles was analyzed before experimentation using dynamic light scattering (DLS) (Supplementary Table 3). Furthermore, particles with surface proteins can exhibit a “sticky” nature, which can cause greater filter fouling over time^64,65^. In this study, membrane fouling (via permeability drop and filtration efficiency) was not observed to be greater with superfolder green fluorescent protein (sfGFP)-coated PSL particles than that with uncoated PSL particles (Supplementary Fig. 8).

**Fig. 5:**
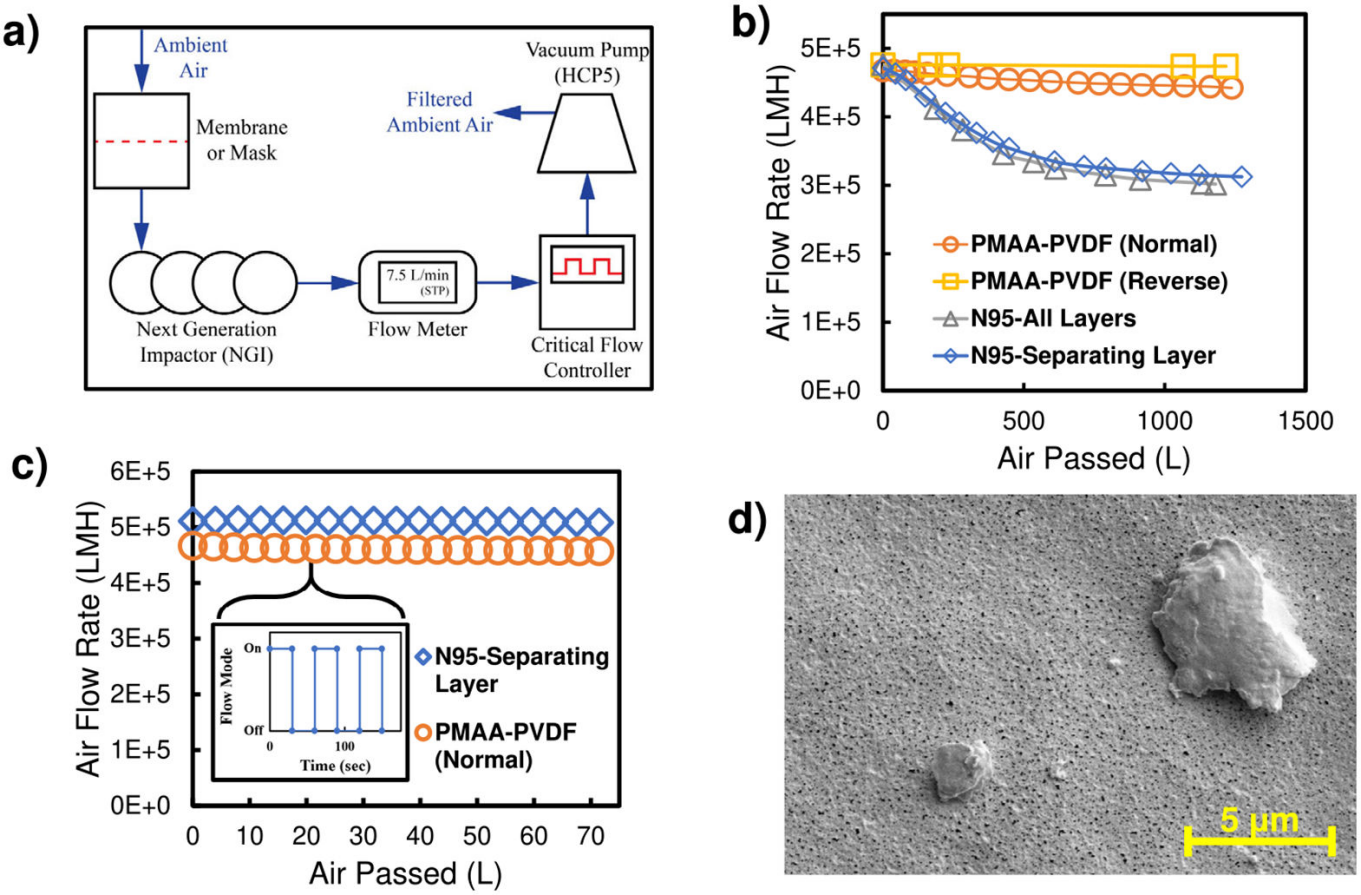
Ambient air filtration through membrane and filter material. **a** Schematic of ambient air-filtration testing of membrane and mask material. Ambient air flux through PMAA-PVDF membrane (normal and reverse orientation), N95 commercial mask (forward orientation), and N95 separating layer (layer 3) under a consistent pressure with **b** continuous flow mode and **c** sinusoidal flow mode of 30 s with flow on and 30 s with flow off (blue diamond and line for N95-separating layer, gray triangle and line for N95-all layers, orange circle and line for PMAA-PVDF-Normal, and yellow square and line for PMAA-PVDF-Reverse). **d** SEM of membrane fouling of PMAA-PVDF membrane after ~500 L of ambient air-filtration. Beginning air flux was set at 7.50 ± 0.50 L/min (average breathing rate). Flow rate measurements normalized at STP. 3 different measurements were taken on the samples (standard deviation resulted in less than 0.5%).

In realistic scenarios, the feed air will not contain PSL particles of similar size, but complex particles of varying size and shape. To test the membrane’s longevity of use as an aerosol filtration material, an ambient air testing module with controlled airflow rate was utilized (Fig. 5a). Using the critical flow controller with continuous flow, the flow rate though PMAA-PVDF and N95 material was set to an average breathing flow rate (calculated from literature)^66^ and was monitored over time when passing ambient air (Fig. 5b). Severe fouling of the N95 mask was observed (33.7% decrease in flow rate after 1273 L of ambient air-filtration), while the PMAA-PVDF membrane showed minimal decrease of 6.3% and 0.4% for normal and reverse orientation, respectively, after about 1200 L of ambient air-filtration. The critical flow controller can run precise sinusoidal-like time frames to mimic inhalation breathing patterns. PMAA-PVDF membranes displayed a slight flow rate drop during sinusoidal on/off cycles (30 s intervals), similar to continuous flow, while the N95-Separating Layer displayed a significantly lower flow drop than that of continuous flow mode (Fig. 5c). This could be attributed to particle dislodgement during breathing patterns, similarly to what occurs during coughing^58^, which could reduce membrane fouling. Ambient particle fouling of membrane was characterized using SEM (Fig. 5d). Furthermore, the void fraction of PVDF400 and PMAA-PVDF membranes were determined experimentally (Supplementary Table 4) to predict the maximum number of 100 nm PSL particles that could be captured with hexagonal lattice or face-centered cubic packing structures (Supplementary Table 5), indicating that the membranes have a high treatment capacity and that PMAA functionalization does not severely lower that capacity.

### Performance of enzyme-functionalized membranes for protein denaturation.

Subtilisin Carlsberg, a stable protease enzyme, could potentially disrupt the spike glycoprotein of SARS-CoV-2. Subtilisin shows high interaction specificity with hydrophobic amino acids that are abundant on the SGP^67^. The rate of enzymatic activity can be quantified using the Michaelis–Menten equation (Eq. 3):

(3) $$v_{{}_{o}}=\frac{V_{\max}[S]}{K_{M}+[S]}$$

where *v_o_* is the reaction rate (M min^−1^ g^−1^), [S] is the substrate concentration (M), *V*_max_ is the maximum reaction rate (M min^−1^ g^−1^), and *K_M_* is the Michaelis-Menten constant (M). Prior to enzyme functionalization, the activity of subtilisin was tested with a known peptide substrate (Supplementary Fig. 9). In a solution-phase reaction, *K_M_* and *V*_max_ values were determined to be 0.25 mM and 0.15 mmol/mg·min at 37 °C, respectively, which agrees with literature^68^.

Enzymatic functionalization of membranes has been commonly researched, both with site-directed and random enzyme immobilization, and can be superior to enzyme solutions in terms of activity maintenance over time, since immobilization reduces interaction of non-selective proteases (e.g., subtilisin) with each other^26,68,69^. PMAA could enhance enzyme loading, as it introduces a charge interaction between the membrane and the enzyme during functionalization. PMAA’s deprotonated carboxylic acid group (pH > pKa value of 4.8^32^) can interact via electrostatic interactions with subtilisin, which is positively charged below its pI of 9.4 (provided by Sigma Aldrich), in solution of 4.8 < pH < 9.4. PVDF400 membranes were first functionalized with PMAA (1.5–3% weight gain of membrane). The PMAA-PVDF membranes were further functionalized with subtilisin in a batch and convective immobilization method (Supplementary Fig. 10a), which was confirmed using Fourier-transform infrared spectroscopy-FTIR (Supplementary Fig. 11). Compared to unfunctionalized PVDF400 membranes, PMAA-functionalized membranes had 84% and 125% higher enzyme loadings during batch and convective functionalization, respectively, confirming that PMAA can enhance enzyme loading and the potential to treat high concentration air sources.

For air-filtration materials, functionalization should not significantly decrease the permeability of the material, as this can harm the breathability of masks and increase the applied pressure need for adequate airflow. The effect of PMAA and subtilisin functionalization on the air permeability of PVDF400 membranes was investigated (Supplementary Fig. 10b). The amount of PMAA and enzyme functionalized can affect the drop in membrane permeability, but, on average, PMAA functionalization decreased the air permeability by 13.4%. Enzyme immobilization decreased the air permeability of the PMAA-PVDF membrane by 8.8% and 29.7% for batch and convective mode, respectively, indicating minor permeability drops with PMAA and enzyme functionalization. The presence of this permeability drop could be mitigated by fabricating PVDF membranes with tailored properties for enhanced airflow, as previously mentioned.

The strength of immobilization between the enzyme and membrane is vital to the long-term performance of the membrane system^70^, especially with random immobilization, which can be less stable than site-specific immobilization. To assess stability, water was passed through Subtilisin-PMAA-PVDF membranes and the permeate was analyzed using ultraviolet-visible spectrophotometry (UV-Vis) until desorption stopped occurring. 0–10% of subtilisin was observed to desorb from the batch-functionalized membrane, indicating that most immobilized enzymes are stable and will not desorb after further membrane usage. This agrees with literature that indicates enzymes that are attached to membrane surfaces via electrostatic interactions show significant stability and negligible enzyme loss^24^. The stability of the functionalized materials indicates that little to no inhalation of enzymes will be present to the mask user.

**Fig. 6:**
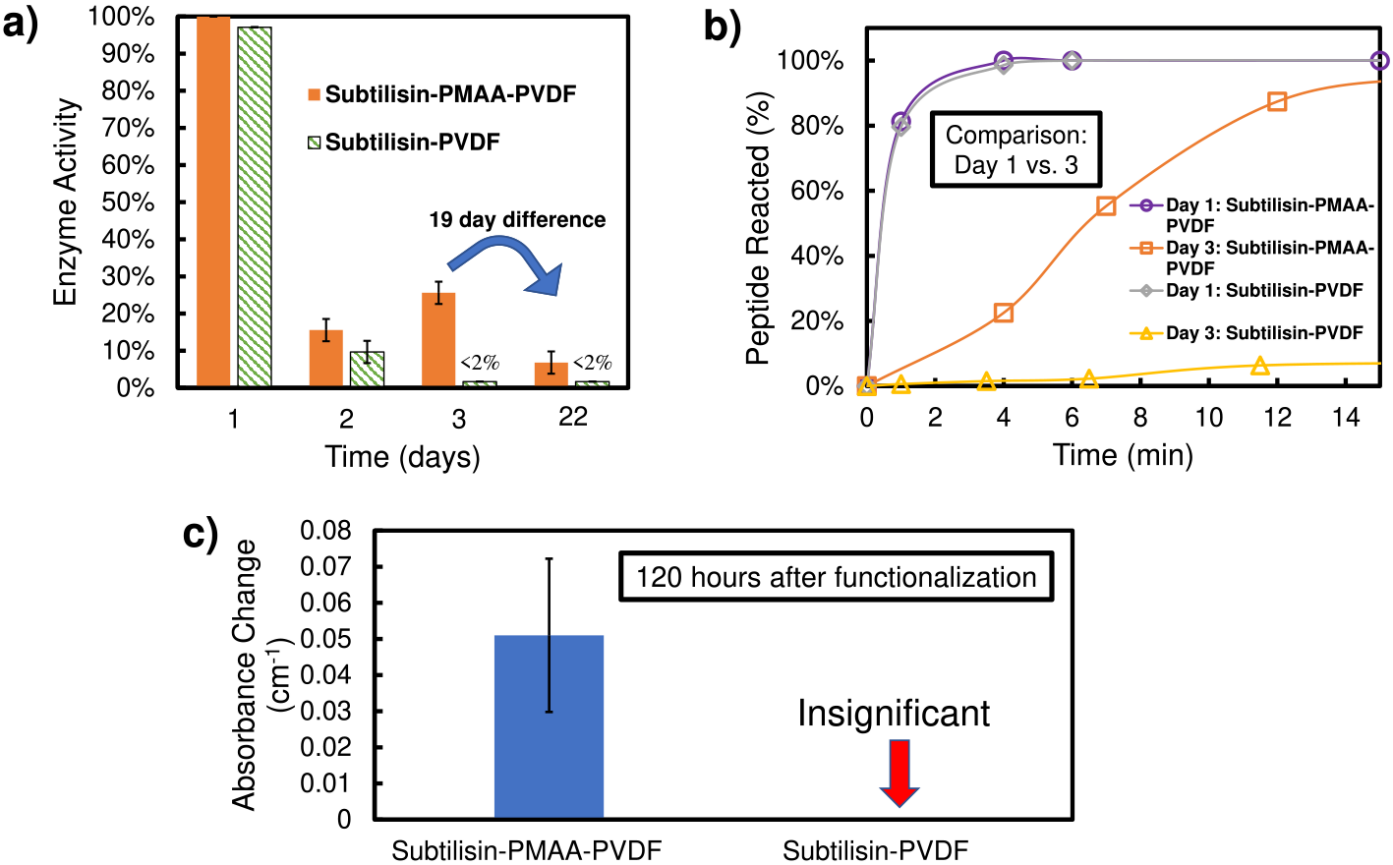
Characterization of enzyme activity immobilized on membrane. **a** Percent of active enzymes immobilized (via batch method) on PMAA-PVDF (orange bar) and unfunctionalized PVDF400 (green striped bar) over time of dry storage with no hydration at 22–24 °C. Day 1 is the day enzyme immobilization occurred. The highest enzyme activity upon immobilization (Subtilisin-PMAA-PVDF) was considered 100% (0.19 mM product formed/min per mg of enzyme) and all subsequent values were normalized based on this activity. **b** Substrate interaction with Subtilisin-PMAA-PVDF (purple circle and line for Day 1, orange square and line for Day 3) and Subtilisin-PVDF membrane system (gray triangle and line for Day 1, yellow triangle for Day 3) 48 h after enzyme functionalization. Peptide concentration was 0.08–0.10 mM. **c** Minimal hydration (37 μL of solution/cm^2^ of membrane = 0.02% water) test of Subtilisin-PMAA-PVDF and Subtilisin-PVDF membranes 120 h after enzyme functionalization. Reaction was allowed to proceed for 60 s. Activity measured using peptide (N-succinyl-Ala-Ala-Pro-Phe-p-nitroanilide) that, upon proteolysis, releases 4-nitroanaline, which absorbs light at wavelength of 410 nm. Initial concentration of peptide was 0.08–0.10 mM and reactions were conducted at 23 °C. Reactions occurred at pH ranging from 7–8. Error bars represent the standard deviation of 3 different measurements taken on the samples.

The structural stability of immobilized enzymes can vary depending on the functionalization method and membrane material^71^, and these enzymes rarely exhibit ~100% of enzyme activity (compared to total active sites). Upon batch immobilization, PMAA-PVDF and PVDF400 membranes had an enzyme activity of approximately 10.2 and 8.2% of equivalent solution-phase activity, respectively. Using solution-phase reaction tests with a known peptide substrate, the experimental and maximum reaction rates were compared to determine the percent of batch-immobilized enzymes active on the membrane over multiple days of dry storage at ambient temperature (~22–24 °C) (Fig. 6a). 48 h after enzyme immobilization (Day 3), subtilisin-functionalized PVDF400 membranes (Subtilisin-PVDF) displayed low enzyme activity (<2% of initial), while Subtilisin-PMAA-PVDF showed ~10% of initial enzyme activity (Fig. 6b). After 3 weeks of dry storage, the Subtilisin-PMAA-PVDF displayed ~7% of initial enzyme activity, which was still greater than that of Subtilisin-PVDF after only 48 h.

Furthermore, these membranes were found to quickly degrade the peptide with minimal hydration (60 s with 0.02% water content) on the sixth day (120 h after enzyme immobilization) (Fig. 6c). The increase in absorbance of 4-nitroanaline (product) at 410 nm for Subtilisin-PMAA-PVDF, compared to no absorbance increase for Subtilisin-PVDF, indicated that the enzyme activity of PMAA-PVDF membranes was significant after days of dry storage and that a high level of hydration is not required for enzyme-substrate interaction. This confirms the hypothesis that the presence of PMAA increases the longevity of immobilized enzymes during dry storage, most likely due to the retention of water in the PMAA hydrogel.

### Deactivation of S-Protein with subtilisin and functionalized membranes.

**Fig. 7:**
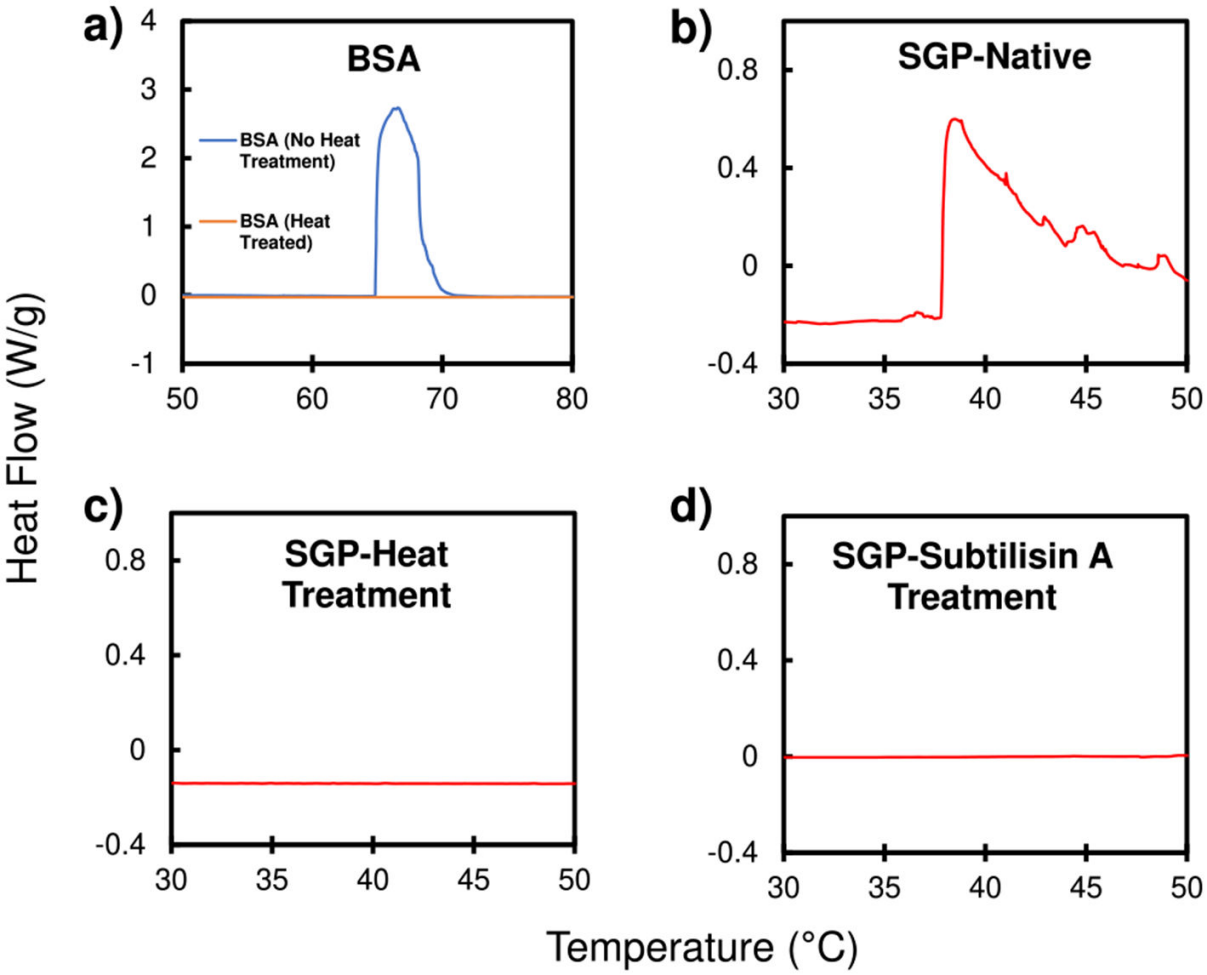
Protein denaturation identified via DSC. DSC thermograph of **a** BSA (10% solution) native (blue line) and heat treatment (orange line) available as reference/standard, SGP solutions (1–2 mg/mL) with **b** no treatment, **c** heat treatment (70 °C for 60 min), and **d** subtilisin treatment (1 mg subtilisin/mL) at a heating rate of 0.5 °C/min (red lines).

Protein denaturation can occur via many methods (pH, thermal treatment, urea treatment), including protease cleavage and simple conformational changes^72^. The SGP of coronaviruses facilitates host cell infection, so denaturation of a portion of these surface proteins would likely reduce the infectivity of the virus by a proportional factor to that of SGP denaturation. Differential Scanning Calorimetry (DSC) can detect protein denaturation^73-75^, as the energy involved in the denaturation process is measured and displayed as a peak in the thermographs. An example of this use of DSC is shown in Fig. 7a, which compares thermographs of Bovine Serum Albumin (BSA), a common protein, before and after heat treatment^76^. In this study, DSC was utilized to determine if subtilisin Carlsberg can denature SGPs of SARS-CoV-2 (Fig. 7b-d). The native SGP thermograph displayed a distinct peak at ~40 °C, indicating that SGP begins to unfold at that temperature. The heat-treated SGP (70 °C for 60 min) displayed no thermograph peak, indicating that the heat denatured the protein prior. Similarly, the enzyme-treated SGP showed no peak, indicating that subtilisin denatured SGP similarly to heat treatment.

**Fig. 8:**
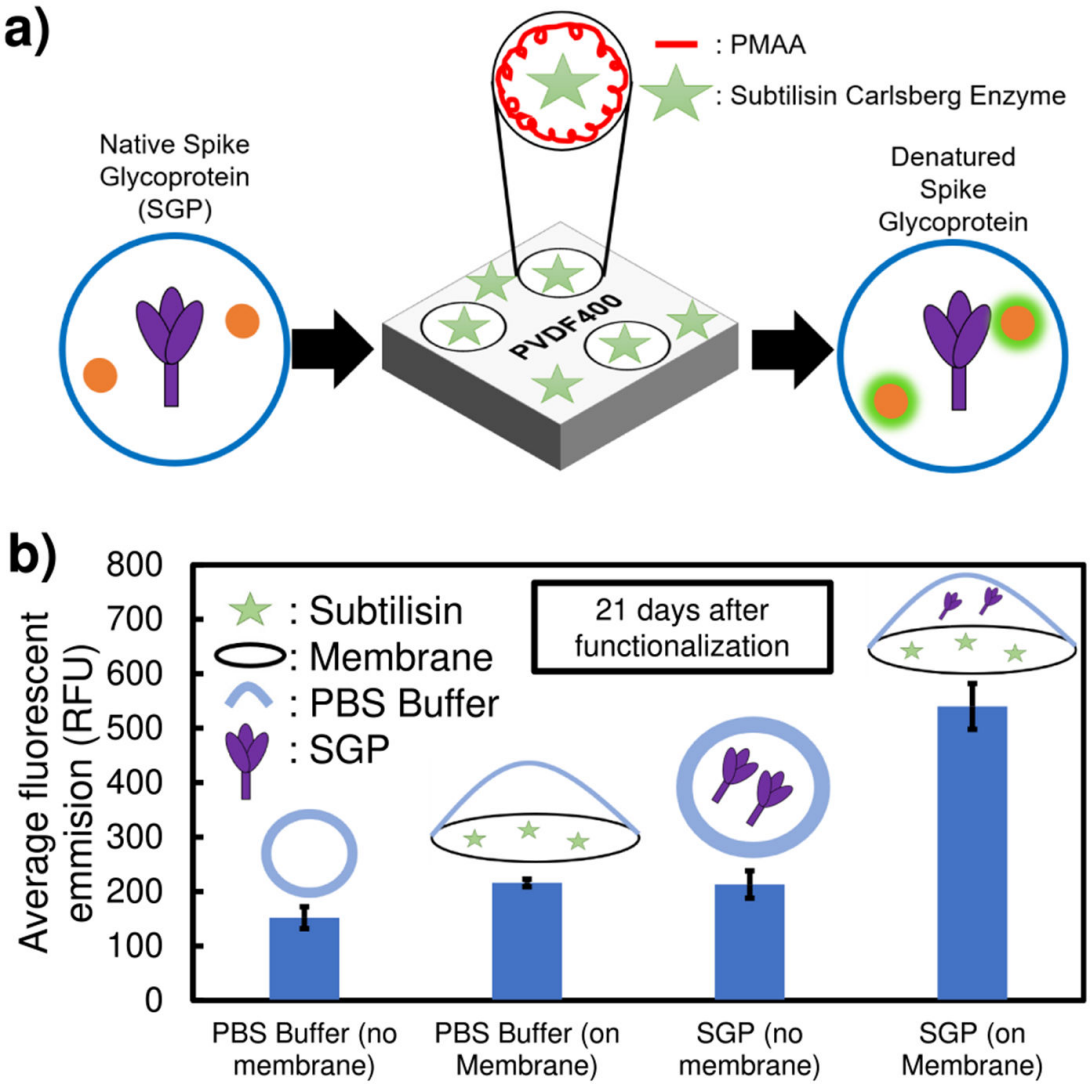
Protein denaturation via enzyme interaction identified using Sypro Orange. **a** Schematic of Sypro-Orange protein denaturation identification method. **b** Average fluorescent emission (RFU) of SGP before and after 30-s exposure to Subtilisin-functionalized (batch) PMAA-PVDF membrane in the presence of hydrophobic-binding fluorescent dye, Sypro Orange. This membrane was functionalized using convective mode and *was left in ambient conditions for 21 days after enzyme functionalization before experiment*. Analyzed using Synergy H1 Hydrid Reader. 0.3 mg/mL of S-Protein and 50x Sypro Orange was utilized. Minimum hydration of membrane with 1.35 μl of solution per cm^2^ of membrane surface during denaturation process (about 0.02 % water content). Reactions were at pH of 7.8 and 23 °C. Error bars represent the standard deviation of 3 different measurements taken on triplicate samples.

Subtilisin has been proven to be stable and interact with substrates in solution-phase. However, when subtilisin is immobilized on an air-filtration material, the presence of water will be limited. Sypro Orange was utilized in this research to determine if Subtilisin-functionalized PMAA-PVDF membranes could denature SGP with minimum hydration (0.02% water content) after long-term exposure to dry conditions (Fig. 8a). Upon denaturation, the protein unfolding exposes the hydrophobic domains that would otherwise be protected inside of the native protein^77,78^. Sypro Orange, a hydrophobic-binding fluorescent dye, has been used for protein denaturation studies^77-79^, and was proven valid in identifying SGP denaturation via change in fluorescent signal intensity. After SGP thermal denaturation (70 °C for 30 min), the Sypro Orange compound showed a 25% increase in fluorescent intensity, showing RFU of 333 and 417 with standard deviations of 32 and 26 for native and thermally denatured, respectively. This increase in fluorescent intensity indicates that the hydrophobic regions of the SGP were exposed to Sypro Orange via heat denaturation and SGP denaturation can be identified utilizing this compound.

A Subtilisin-PMAA-PVDF membrane was functionalized via convective mode and left in ambient dry conditions for 21 days before this experiment (no hydration at 23 °C) to test long-term realistic storage scenarios. When SGP was placed on three different points of the enzyme-functionalized membrane, an increase in average fluorescent intensity of SGP was observed relative to SGP on a membrane with no enzyme (Fig. 8b). The goal of this process was to simulate the deposition of small hydrated aerosol droplets on a mask surface. This statistically-significant finding (Supplementary Table 6) indicated that membrane-bound subtilisin could denature SGP in low-humidity filter applications and does not require a high humidity scenario after multiple weeks of dry storage.

**Fig. 9:**
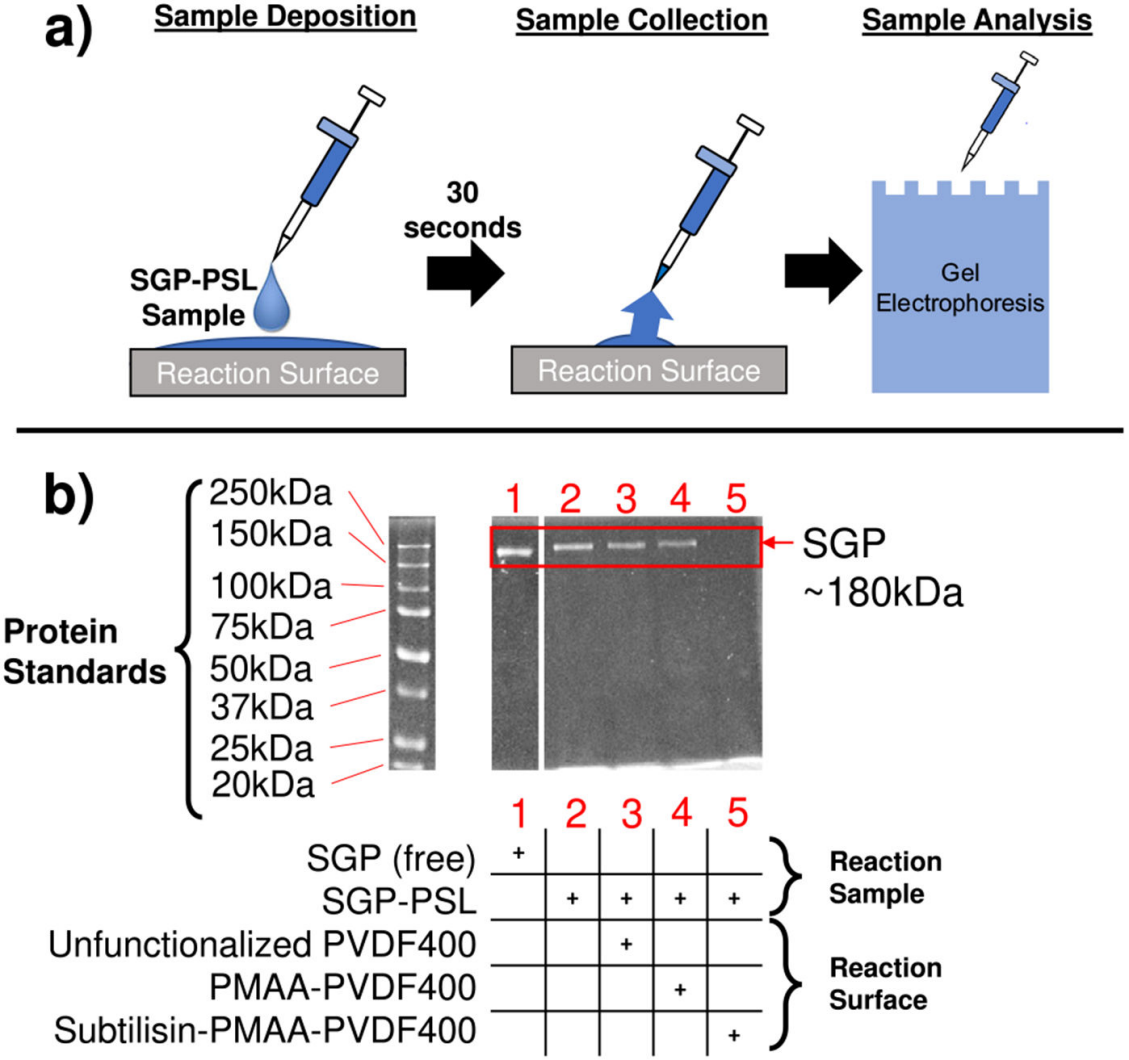
Characterization of particle-bound protein denaturation via enzyme interaction of functionalized membrane. Analysis of 100 nm SGP-functionalized PSL after **a** 30 s reaction with different membrane surfaces via **b** Sodium dodecyl-sulfate polyacrylamide gel electrophoresis (SDS-PAGE). Approximately 2.6 μg of protein was loaded in each well (except the SGP-only lane with ~5.2 μg) with reactions carried out at total protein concentrations of ~87.5 μg/mL at pH of 7.8 and 23 °C. These experiments were performed in duplicate (see Supplementary Fig. 12 for the complete gel image and the second experiment data).

In realistic scenarios, spike glycoproteins will be bound to the coronavirus particle surface when exposed to the enzyme-functionalized membrane, thus only protein that physically encounters the enzymes will be denatured. To investigate the degree of surface-bound protein that is denatured, SGP-functionalized PSL particles (SGP-PSL) were produced to mimic real coronavirus particles and reacted on enzyme-functionalized membranes at similar minimum humidity conditions to that of the SGP solution tests (Fig. 9a). After reaction with subtilisin-PMAA-PVDF membranes for 30 s, free spike glycoprotein and SGP-PSL samples analyzed using gel electrophoresis showed a complete disappearance of full-length SGP (~180 kDa) present in the control samples (Fig. 9b). This shows that all spike glycoprotein (above the limit of detection) on coronavirus-sized particles interacted with the enzymes on the PVDF membrane, indicating that the developed membrane system could substantially deactivate the coronavirus’s ability to infect its host (via loss of surface-bound full-length spike proteins). See Supplementary Fig. 12 for the complete gel image and the second experiment data.

## Discussion

For virus particle filtration, water membranes have a distinct energy advantage in air-phase filtration, as the minimum pressure to obtain airflow through the membrane is low, while that for water flow of hydrophobic membranes is significantly higher. For PVDF Durapore membranes, the hydrophobic membrane did not exhibit water flux until ~3.5 bar (breakthrough pressure), while the hydrophilic did at <0.7 bar (Supplementary Fig. 13a), indicating the increased pressure need to conduct water-filtration with hydrophobic material. The minimum pressure to obtain water flux can be calculated using the Young–Laplace Equation:

(4) $$P_{cap}(bar)=\frac{4\gamma_{wo}*\cos(\theta )*10^{-5}}{d_{p}}$$

where *P*_*cap*_ is the capillary or breakthrough pressure (bar), *γ*_*wo*_ is the surface tension of the liquid interface (N per m), *d_p_* is the effective membrane pore diameter (m), and *θ* is the contact-angle (°).

Using literature-estimated surface tension values, the *P*_*cap*_ of the hydrophobic and hydrophilic membrane were calculated to be −3.3 (*P*_*cap*_ < 0) and 0.46 (*P*_*cap*_ > 0), respectively, for the largest pores identified on SEM, which agrees with experimental results and literature^80^. Air-filtration was achieved through both hydrophilic and hydrophobic membranes with miniscule breakthrough pressures (<0.01 bar) (Supplementary Fig. 13b), confirming that virus membrane filtration can yield a lower energy-cost in air-filtration than water-filtration. Given that the hydrophilicity of filtration surfaces can affect both virus deactivation after capture^81^ and fouling during filtration of hygroscopic particles^82^, we investigated surface characteristics of membranes during our initial survey (Supplementary Fig. 13c).

Models of compressible fluid flow through membrane pores must account for fluid density, which changes depending on the pressure gradient present. When investigating the use of Eq. 2 for airflow through an Isopore Polycarbonate membrane, the calculated Knudsen number (4.8 × 10^−4^) is very small, indicating a negligible effect on flow through similar membrane systems, including PVDF400. Polycarbonate Isopore membranes were used for airflow modeling and calculations because of simplifications in the calculations (tortuosity = 1) and because polycarbonate membranes have been studied by the CDC for aerosol capture^54^. The mass flowrate through a single Isopore membrane pore (~200 nm diameter) at pressure difference (ΔP) of 0.08 bar found using Eq. 2 and experimental measurements were 3.50 × 10^−14^ kg/min and 9.24 × 10^−14^ kg/min, respectively. For PVDF400 (only PVDF layer), the values were 1.72 × 10^−16^ kg/min and 1.29 × 10^−15^ kg/min, respectively. Estimations from Eq. 2 are less accurate for PVDF400 than for Isopore membranes because the equation does not account for tortuosity; also, PVDF400 has a wider pore-size distribution than the Isopore membrane. Overall, these results indicate that this model is appropriate for modeling low-pressure compressible fluid flow through uniform membrane pores and that the equivalent global membrane variable parameter (including porosity, pore radius, and thickness) is appropriate for the design of membrane variables that match air permeability of commercial face masks.

As previously stated, filters have three particle interaction modes. From Fig. 5, the main interaction mode for PVDF400 membranes is presumably sticking with minimal penetration when using ~100 nm PSL particles, as SEM imaging showed particle adherence to the membrane surface, and particle penetration through the membrane is low (high filtration efficiency). This experiment was done under applied air pressure, so these conclusions are valid for high-pressure filter systems. For mask application with lower pressures, the splash/rebound mode could be more common than sticking, especially with in/out breathing flows, as there is not enough pressure to force particle sticking, but this mode would still be minimal for the functionalized membranes, due to the membrane hydrophilicity. Mask material is typically hydrophobic to allow a high material drying rate^83^, which results in lower virus transmission rate^84^. The hydrophilicity of Subtilisin-PMAA-PVDF membranes allows more water to soak in the membrane surface pores, which could translate to enhanced enzymatic performance under low-humidity conditions.

The duration of effective use for respiratory masks is relevant to the protection duration of filters and the amount of material sent to landfills. Figure 5 can be utilized to estimate the longevity of PMAA-PVDF membrane use in a mask application with exhalation valve (inlet flow only). With an average breathing rate of 18 breaths/min^85^, a 70-kg individual with an average tidal volume of 500 mL/breath could wear a PMAA-PVDF mask for several days (2 h of daily use) without a significant drop in breathability, while N95 masks could become difficult to use after one day. As membrane-based masks could be used for longer periods than N95 options before disposal is required, the environmental impact of personal protective equipment (PPE) disposal and subsequent plastic pollution could be reduced significantly, especially during periods of increased PPE usage, such as pandemics^86^. Furthermore, the membrane permeability drop using ambient air was lower than that of PSL aerosols (2209 and 19,000 aerosol particles/L in 0.3–0.5 μm range, respectively), most likely due to the difference in particle count of smaller particles that can penetrate inside the pore and foul/clog the membrane.

The protection factor (PF) of a mask material is a common quantification of protection for the individual (used by CDC and Occupational Safety and Health Administration-OSHA); specifically, it is calculated as the ratio of inlet concentration to outlet concentration (C_feed_/C_permeate_). OSHA has designated the PF of approved N95 masks to be >10, which was confirmed by Lee et al. who found PFs of N95 masks to range from 10–100 for 0.3–0.5 μm aerosol particles^6^. Based on our results, PVDF400 has a PF of 540 ± 380 for aerosols of 0.3–0.5 μm size, indicating that this membrane, on average, surpasses the protection offered by N95. This allows for high flexibility and freedom of membrane design (e.g., thickness reduction) to improve airflow while still meeting or exceeding the performance of N95 masks. Furthermore, the cost of a single functionalized membrane mask was estimated to be $0.63 (based on material costs), while the purchase cost of commercially-available surgical and N95 masks are roughly $0.14 and $1.31, respectively. Despite a low material cost, the functionalized membrane still exhibits a significantly higher protection factor than surgical and N95 masks (Supplementary Fig. 14), making the proposed thin-film membrane system superior in terms of protection, while still remaining comparable in terms of cost to existing PPE options.

The hydration of subtilisin plays a vital role in the enzyme stability, which could explain the different activity results between PMAA-PVDF400 and PVDF400 membranes over time in Fig. 6. On Day 1, the hydration of the enzymes is most prominent, as the membrane has not been left in dry storage yet, thus the activity is highest. Though, after multiple days of dry storage, the re-hydration of the enzymes will not be instantaneous, especially due to (1) batch mode activity test that does not use a pressure gradient to move substrate through membrane (not exposing all enzymes immediately) and (2) PMAA functionalization could maintain structure and activity better, as well as increasing speed of re-hydration, of enzymes directly exposed to the polymer. The importance of PMAA-functionalization on enzyme hydration is further emphasized in the significantly higher reaction rate of Subtilisin-PMAA-PVDF, compared to Subtilisin-PVDF in Fig. 6b. In realistic scenarios, the membrane’s minimum hydration required to maintain enzyme activity should be met from respiratory water loss of human user (31 mg H_2_O/Liter of air exhaled^87^) and maintained by the PMAA polymer. Overall, despite the lack of consistent hydration, enzyme activity for hydrophilized PMAA-PVDF membranes still remains significant (>10% of initial) and produces 100% of substrate product, simply with longer reaction times compared to the initial activity reaction time.

## Conclusion

This paper investigated the development of a membrane-based respiratory face mask and enclosed-environment filter that can denature the spike glycoproteins of coronaviruses, specifically SARS-CoV-2, and lead to virus deactivation. PVDF400 water-filtration membranes were evaluated and compared to existing PPE options via three main criteria: (1) protection factor (ratio of inlet concentration of particles to outlet), (2) fouling of material by aerosol particle contamination and subsequent permeability decrease, and (3) size distribution of permeate aerosolized particles. These membranes can be applied to air-filtration uses and offer high protein-functionalized aerosol filtration efficiency and capture (with greater longevity of usage) than commercial N95 masks. PVDF membranes are also expected to exhibit high breathability with a specified porosity, pore size, and thickness. Subtilisin-functionalized PMAA-PVDF membranes, in turn, can enhance protection from viral infection via denaturation of SGP with minimal membrane hydration, indicating that this work significantly advances enzymatic reaction science on hydrophobic/hydrophilic surfaces with minimal hydration. In low humidity environments, Subtilisin-functionalized PMAA-PVDF membranes have been proven to be a promising system of advancement towards the new generation of respiratory face masks and enclosed-environment filters that can significantly reduce coronavirus transmission by virus protein deactivation and enhanced aerosol particle capture.

## Materials and methods

### Contact-angle.

The contact-angle was measured for membrane samples over time. The sessile-drop method was selected for contact-angle measurements (Drop Shape Analyzer-DSA 100, KRÜSS Scientific Instruments, Inc.). This method was carried out by manually drawing a 3 μL DI water droplet (pH ~6) and using the instrument to deposit the drop onto the membrane surface. Data collection was started (time = 0) immediately after the deposition of the drop. Triplicate data were collected every 2 s for up to 84 s following the drop deposition. Drops with contact-angles above 20° were fitted using the Young Laplace method while drops with contact-angles below 20° were fitted as circles.

### Void fraction.

The measurement of solvent uptake by the membranes was used to determine their void fraction. PVDF400 membrane samples were cut into disks with an area of 19.6 cm^2^; multiple thickness measurements on each of these samples were taken with a micrometer to allow for the calculation of the membrane volume. ISOPAR-G was selected as the solvent because it was observed to thoroughly wet PVDF400 membranes. Membrane samples were weighed, soaked in isoparaffinic hydrocarbon fluid (ISOPAR-G) for about 2 h, and then weighed again. Porosity values from each trial could then be calculated using Eq. 5.

(5) $$\phi =\frac{m_{\text{solv}}∕\rho_{\text{solv}}}{A_{\text{mem}}*\delta_{\text{mem}}}$$

where m_solv_ is the difference in mass between the wet and dry membranes, ρ_solv_ is the solvent density, A_mem_ is the area of the membrane surface, and δ_mem_ is the measured thickness of the membrane. Supplementary Table 2 shows the calculated void fractions and pore volumes (m_solv_/ρ_solv_) for PVDF400 and PMAA-PVDF membranes.

### PMAA functionalization of PVDF.

PMAA was introduced into the commercial PVDF400 membranes using a technique similar to one detailed previously for the creation membranes containing PAA or PMAA^26,30,88^. A polymerization solution in deoxygenated water (pH of 5.3–6.5) was created from methacrylic acid (MAA; monomer, 0.06–0.15 weight fraction of membrane), N,N’-Methylenebisacrylamide (MBA; crosslinker, 1 mol% relative to MAA), and potassium persulfate (KPS; initiator, 1 mol% relative to MAA). Using vacuum filtration, solutions were drawn through the membrane multiple times through the top and back of the membrane. Membranes were then wrapped in a plastic wrapping, clamped between Teflon plates, and heated at 80° under a vacuum (P ~0.6–0.7 bar vacuum) for 1.5–1.7 h. After this time, the wrapped membranes were removed from between the plates and heated again under vacuum or at atmospheric pressure for ~30 min. Lastly, membranes were removed from the plastic wrappings and placed in the oven under vacuum or at atmospheric pressure for 15–30 min. A final mass value was compared to the initial mass to determine the amount of PMAA added to each membrane. For membrane and pore functionalization, potassium persulfate (Acros Organics, CAS: 7727-21-1), methacrylic acid (stab. with 250 ppm 4-methoxyphenol, Alfa Aesar, CAS: 79-41-4), and N,N’-Methylenebisacrylamide (Alfa Aesar, CAS: 110-26-9) were purchased. Full-sized commercial polyvinylidene fluoride microfiltration membranes (PVDF400, porosity: 0.46, thickness: 165 μm, average pore diameter: 45 nm with ~5% of pores having a diameter of 100–120 nm) were provided by Solecta, Inc., Oceanside, CA.

### Membrane filtration (water and air) and mask testing.

Dry-air-filtration experiments were conducted using applied pressure from compressed extra dry grade air (Catalog Number: 11, American Welding & Gas). The humidity of the compressed dry-air was confirmed using an in-line wireless humidity sensor (SensoNODE Blue-Parker). Polycarbonate in-line filter holders (In-Line Filter Holders, 47 mm, Pall Laboratory) and custom-made Honeywell Stainless Steel cells were used as air membrane/mask filtration cells. For water-filtration, dead-end stirred Millipore cells were used. Airflow rate through the membrane was measured at various applied pressures using a digital in-line flowmeter (TSI 4043) to calculate membrane permeability. For commercial membranes, airflow rate experiments were conducted in normal and reverse orientations. For commercial masks, airflow rate experiments were conducted in a normal orientation as well, meaning that the side of the mask that would be exposed to the ambient environment was the air inlet side, while the side of the mask that would be exposed to the human’s mouth and nose was the air outlet side. The pressure drop (ΔP) across the membranes/masks were measured using a pressure manometer (Catalog Number: 33500-086, Manometer Pressure/Vacuum Gauge-VWR International) connected directly before and after the filter holder cell. Hydrophilized PVDF Durapore membranes were purchased from Millipore Sigma (100 nm: VVLP09050, 220 nm: GVWP09050). Polycarbonate Isopore membranes (diameter~100 nm) were purchased from Millipore Sigma (GTTP04700). NIOSH-approved N95 masks were purchased from Fastenal (SKU: 1049938) and surgical masks were purchased from Yantai Fushuntai Biotechnology Co. Polysulfone ultrafiltration membranes (PS35, porosity: 0.035, thickness: 224 μm, average pore diameter: 15–20 nm) were provided by Solecta, Inc., Oceanside, CA. and thin-film composite nanofiltration membranes (DOW-FilmTec NF270, thickness: 152 μm, average pore diameter: 0.8 nm).

Membrane water and air permeability was calculated by the equation below:

(6) $$Membrane\;Permeability=\frac{Q}{A\times \Delta P}$$

Where *Q* is volumetric flow rate (L/min), *A* is membrane area (m^2^), and *ΔP* is transmembrane pressure drop (bar).

### Membrane aerosol filtration by particle size.

PSL-based aerosols were generated using the TSI 3076, a collision-type aerosol generator that introduces PSL from a solution to a polydisperse aerosol (Supplementary Fig. 15). 2.1 bar of applied pressure was fed into the generator for aerosolization. The size of PSL particles were quantified using a dynamic light scattering particle analyzer (Litesizer 500-Anton Paar). For permeate size distribution readings, 3 mL samples of the permeate were taken after bubbled into DI water and size measurements were volume-weighted with confirmed baseline within 1.000 ± 0.01. A run time of 10 min was allowed to ensure adequate capture of aerosolized particles in the bubbled water. Unfunctionalized polystyrene latex nanoparticles (average diameter: 100 nm) were purchased from Sigma Aldrich (LB1).

### Membrane aerosol capture, filtration efficiency, and permeability drop via fouling.

PSL-based aerosols were generated using the TSI 3076, a collision-type aerosol generator that introduces PSL from a solution to a polydisperse aerosol. The aerosol feed solution was sonicated for 5 min and then placed in the atomizer feed reservoir and sealed. Initially, only filtered dry-air was used to flush the system for 10–15 min, then the atomizer air feed was also turned on and the system was allowed to equilibrate for 10–15 min. The particle count, flow, and pressure drop measurements were taken with this equilibrated aerosol stream as initial starting point data before switching the bypass valve to initiate filtration of the air stream. After 1 min equilibration with the filter inline, particle counts, flow rate, and pressure drop measurements were taken as the initial timepoint for the filtered stream. Immediately following the filtered air data collection, the bypass valve was switched to obtain measurements for the unfiltered air stream at the corresponding time point in the same manner after 1 min equilibration. Met-One Instruments’ GT-526S particle counter was used to count concentration of PSL aerosols in air, categorized into different particle sizes. The membrane permeate after aerosol feed flow was captured in a closed-off module to allow for accurate particle count (Supplementary Fig. 16).

Filtration efficiency was calculated by the equation:

(7) $$Filtration\;Efficiency(\%)=\frac{C_{F}-C_{P}}{C_{F}}\times 100$$

where *C_F_* is particle concentration of the feed air stream (particles/L) and *C_P_* is particle concentration of the permeate stream (particles/L).

### sfGFP and SGP functionalization of COOH-PSL particles.

sfGFP and SGP functionalization of PSL particles with COOH groups (COOH-PSL) was adapted from literature^89^. Briefly, a suspension (10.2% solids) of 100 nm nominal diameter polystyrene latex nanoparticles (PSL-NPs) with carboxylate surface functionalization (Bangs laboratories) were washed twice by 20x dilution and then diluted 10–20x into 10 mM NiCl_2_. After 1 h of incubation, the Ni+ labeled PSL-NPs were diluted~10–100x into a 500 nM and 0.08 mg/mL solution of polyhistidine-tagged superfolder GFP and spike glycoprotein, respectively, in 1x Phosphate Buffered Saline (PBS) at pH of 8.0 followed by ~60–90 min incubation at room temperature. The protein-functionalized particles were then washed three times with a final dilution to ~0.5% and ~2.5% solids content of PSL-sfGFP and SGP-PSL, respectively, in 1xPBS (pH = 8.0). The suspension was stored protected from light for less than a week at 4 °C before usage in aerosol experiments. COOH-functionalized polystyrene latex nanoparticles were purchased from Bangs Laboratories (PC02004). Green fluorescent protein (GFP) with 6-histidine tagging at the N-terminus was made in Dr. Yinan Wei’s lab from the Department of Chemistry at the University of Kentucky. Nickel (II) chloride hexahydrate was purchased from Sigma Aldrich (7791-20-0).

### Enzyme solution-phase reaction.

The activity of the subtilisin enzyme was tested in a solution-phase with a peptide (N-succinyl-Ala-Ala-Pro-Phe-p-nitroanilide) that, upon proteolysis, releases 4-nitroanaline, which absorbs light at wavelength of 410 nm. Initial substrate concentration was 0.8 mM with an enzyme concentration of 0.12 mg/L. The reaction was conducted at 37°C and a consistent pH of 7.8. pH was maintained by using a 50 mM Phosphate buffer. Activity was characterized with calculated values of *K*_M_ and *V*_max_. Wavelength readings were obtained using the UV-6300PC Double Beam Spectrophotometer. Subtilisin Carlsberg was purchased from Sigma Aldrich (P5380) and N-Succinyl-Ala-Ala-Pro-Phe p-nitroanilide was purchased from Sigma Aldrich (S7388). 4-nitroanaline (to make standard curve for concentration analysis to test enzyme activity) was purchased from Millipore Sigma (185310).

### Ambient air-filtration.

The membrane/mask was placed in a filter holder (In-Line Filter Holders, 47 mm, Pall Laboratory) for the airflow inlet and airflow was generated using a vacuum pump (HCP5-Copley). A Next Generation Impactor (NGI) was included to mimic human lung pressure drop. A steady airflow rate of approximately 7.5 L/min was initially set using a critical flow controller (TPK2000-Copley) at a temperature of 23.5 °C and the decrease in airflow through the membrane/mask was monitored using the digital flow meter. Sinusoidal on/off flow rate testing was done over 1-minute intervals (30 s with flow on, 30 s with flow off). Images of filtration cells used for experimentation are available in Supplementary Fig. 17.

### Membrane functionalization with enzyme.

Both PVDF400 and PMAA-PVDF membranes were convectively immobilized with subtilisin Carlsberg. For batch immobilization mode, 100 mL of 0.1 mg/mL solution of subtilisin was stirred in a water-filtration cell for 60 min. For convective immobilization mode, membranes were compacted once with a basic solution of sodium hydroxide in deionized water (pH 9) at 1 mL/min for 60 min and then again with deionized water at 1 mL/min for 60 min. The membranes were rinsed and immobilized with a 0.1 mg/mL solution of subtilisin Carlsberg at approximately 0.67 ml/min for 60 min. Mass of enzyme immobilized was determined by analyzing the subtilisin concentration of the functionalizing solution before and after the immobilization process with the UV-6300PC Double Beam Spectrophotometer (VWR) at a wavelength of 280 nm. Membranes were stored in “dry storage” conditions, meaning in a dry unsealed petri dish in a controlled-environment room (exposed to light) with a relative humidity of 40–60% and temperature of 15–21 °C.

### SGP denaturation (heat, enzyme) identified via Dynamic Scanning Calorimetry (DSC).

The confirmation of the proteins (denatured versus native) was determined by obtaining thermographs of protein solutions using a dynamic scanning calorimeter (DSC-Q200 by TA Instruments). The existence of a thermograph peak (temperature value confirmed by literature) indicated that the protein was active/folded before DSC analysis, while the absence of a specific peak indicated that the protein was denatured before DSC analysis via a denaturing treatment method. BSA protein solutions were initially tested to determine if this method was appropriate for determining protein denture by comparing the thermographs of a stock BSA solution with a heat-treated BSA solution. The heat treatment of BSA consisted of heating the solution to 90 °C for 60 min. SGP solutions of 1–2 mg/mL were tested to determine if subtilisin denatures the protein in a similar manner as heat or alcohol treatment does. The thermograph of the SGP stock solution was compared to thermographs of a heat-treated SGP solution, a subtilisin-treated SGP solution (1 mg subtilisin/mL), and an alcohol-treated SGP solution (50% alcohol). Heat treatment of SGP was conducted at 70 °C for 60 min. The lower limit of protein concentration in the sample is 0.5 mg/mL.

### SGP denaturation (heat) identified via Sypro Orange.

For thermal denaturation, SGP in 20 mM HEPES (4-(2-hydroxyethyl)–1-piperazineethanesulfonic acid)) buffer was heated at 70 °C for 30 min. After the heating process, the solution was allowed to return to ambient temperature (23 °C) and was then combined with Sypro Orange. The heated sample was compared to an unheated sample of SGP combined with Sypro Orange in the same HEPES buffer. For each sample, 6 μL of 0.3 mg/mL SGP was combined with 30 μL of 20 mM HEPES buffer and 4 μL of 50X Sypro Orange solution. The fluorescent intensity was measured using a BioTek Synergy 96 well plate reader. 40 μL total volume was used with an excitation and emission wavelength of 483 nm and 568 nm, respectively, at 25 °C.

### Denaturation of free and particle-bound SGP on enzyme-functionalized membrane (minimal hydration).

Sypro Orange, a dyhe that produces a fluorescent signal upon exposure to a hydrophobic environment and is commonly used for determining protein denaturation, was utilized to identify free SGP denaturation. Subtilisin Carlsberg, a commonly used non-selective serine protease, was used to denature SGP. Membrane-bound subtilisin was used such that SGP could be introduced in a liquid drop onto the surface of the membrane and interact with the enzyme with minimum water presence, mimicking semi-dry mask conditions. Four different samples were made. Sample 1: 6 μL of 50X Sypro Orange solution (stock 5000X concentrated in DMSO) in 34 μL of 1X PBS buffer. Sample 2: 6 μL of 50X Sypro Orange solution in 34 μL of 1X PBS buffer that had been placed on a Subtilisin-PMAA-PVDF membrane for 30 s. Sample 3: 4 μL of 0.3 mg/mL S-Protein (in PBS) that had not contacted subtilisin or membrane, 6 μL of 50X Sypro Orange solution, and 30 μL of 1X PBS buffer. Sample 4: 4 μL of 0.3 mg/mL S-Protein (in PBS) that had been placed on a Subtilisin-PMAA-PVDF membrane for 30 s, 6 μL of 50X Sypro Orange solution, and 30 μL of 1X PBS buffer. Sypro Orange fluorescent gel dye was purchased from Sigma Aldrich (S5692). Full length trimeric His6-tagged spike protein’s codon-optimized cDNAs for mammalian expression and its His6-tagged receptor binding domain were kindly provided by Dr. Florian Krammer of the Icahn School of Medicine at Mount Sinai, New York. For protein stabilization, the full-length spike protein was modified (furin cleavage site removal) by the Krammer lab. These cDNAs were expressed in Expi293 cells and purified by Ni^2+^ affinity chromatography. Details of methods and scientific background are provided by Stadlbauer et al.^90^.

Denaturation of SGP bound to PSL particles was identified using SDS-PAGE. First, particle reaction was conducted via 50 μL droplets of 0.08 mg/mL SGP and 2.5% solids content onto surface of batch-mode subtilisin-PMAA-PVDF400 membrane for 30 s. Samples were then prepared by boiling with reducing SDS loading dye for 5 min before loading in the wells of 10% SDS-polyacrylamide gels (4% stacking/10% resolving) for electrophoresis to analyze degradation of SGP by subtilisin-membrane treatment, followed by Coomassie total protein staining and imaging (BioRad ChemiDoc MP).

## Supplementary Material

Supplementary Material

## Data Availability

The data that support the findings of this study are available from the corresponding author upon reasonable request.

## References

[1] Sills J Airborne transmission of SARS-CoV-2. Science 370, 303–304 (2020). 10.1126/science.abf0521 33020250

[2] Zhou L, Ayeh SK, Chidambaram V & Karakousis PC Modes of transmission of SARS-CoV-2 and evidence for preventive behavioral interventions. BMC Infect. Dis 21, 496 (2021). 34049515 10.1186/s12879-021-06222-4 PMC8160404

[3] Cheng Y Face masks effectively limit the probability of SARS-CoV-2 transmission. Science 372, 1439–1443 (2021). 10.1126/science.abg6296 PMC8168616 34016743

[4] Chua MH Face masks in the new COVID-19 normal: Materials, testing, and perspectives. Research 2020, 7286735–7286735 (2020). 32832908 10.34133/2020/7286735 PMC7429109

[5] Yim W KN95 and N95 respirators retain filtration efficiency despite a loss of dipole charge during decontamination. ACS Appl. Mater. Interfaces 12, 54473–54480 (2020). 33253527 10.1021/acsami.0c17333 PMC7724761

[6] Lee SA, Grinshpun SA & Reponen T Respiratory performance offered by N95 respirators and surgical masks: human subject evaluation with NaCl aerosol representing bacterial and viral particle size range. Ann. Occup. Hyg 52, 177–185 (2008). 18326870 10.1093/annhyg/men005 PMC7539566

[7] Thomas R, Guillen-Burrieza E & Arafat HA Pore structure control of PVDF membranes using a 2-stage coagulation bath phase inversion process for application in membrane distillation (MD). J. Membr. Sci 452, 470–480 (2014).

[8] Wang L-Y, Yu LE, Lai J-Y & Chung T-S Developing ultra-high gas permeance PVDF hollow fibers for air filtration applications. Sep. Purif. Technol 205, 184–195 (2018).

[9] Leung WW-F & Sun Q Charged PVDF multilayer nanofiber filter in filtering simulated airborne novel coronavirus (COVID-19) using ambient nano-aerosols. Sep. Purif. Technol 245, 116887–116887 (2020). 32372877 10.1016/j.seppur.2020.116887 PMC7194611

[10] Palika A An antiviral trap made of protein nanofibrils and iron oxyhydroxide nanoparticles. Nat. Nanotechnol, 10.1038/s41565-021-00920-5 (2021). 34083772

[11] Hewawaduge C, Senevirathne A, Jawalagatti V, Kim JW & Lee JH Copper-impregnated three-layer mask efficiently inactivates SARS-CoV2. Environ. Res 196, 110947 (2021). 33662346 10.1016/j.envres.2021.110947 PMC7919544

[12] Rubino I Salt coatings functionalize inert membranes into high-performing filters against infectious respiratory diseases. Sci. Rep 10, 13875 (2020). 32807805 10.1038/s41598-020-70623-9 PMC7431535

[13] Barati F, Hosseini F, Habibi Moghadam F & Abbasi Dezfouli S Face mask as a tool to prevent the Coronavirus disease 2019: The importance and challenges. 7, e109729, (2021).

[14] Betts MJ & Sternberg MJE An analysis of conformational changes on protein–protein association: Implications for predictive docking. Protein Eng. Des. Sel 12, 271–283 (1999). 10.1093/protein/12.4.271 10325397

[15] Saravanan KM & Selvaraj S Dihedral angle preferences of amino acid residues forming various non-local interactions in proteins. J. Biol. Phy 43, 265–278 (2017). 10.1007/s10867-017-9451-x PMC5471173 28577238

[16] Gong H, Fleming PJ & Rose GD Building native protein conformation from highly approximate backbone torsion angles. Proc. Natl Acad. Sci. USA 102, 16227 (2005). 16251268 10.1073/pnas.0508415102 PMC1283474

[17] Privalov PL Heat capacity and conformation of proteins in the denatured state. J. Mol. Biol 205, 737–750 (1989). 2538636 10.1016/0022-2836(89)90318-5

[18] Gordon DE A SARS-CoV-2 protein interaction map reveals targets for drug repurposing. Nature 583, 459–468 (2020). 32353859 10.1038/s41586-020-2286-9 PMC7431030

[19] Naqvi AAT Insights into SARS-CoV-2 genome, structure, evolution, pathogenesis and therapies: Structural genomics approach. Biochimica et Biophysica Acta (BBA) - Mol. Basis Dis 1866, 165878 (2020). 10.1016/j.bbadis.2020.165878 PMC7293463 32544429

[20] Astuti I & Ysrafil Severe Acute Respiratory Syndrome Coronavirus 2 (SARS-CoV-2): An overview of viral structure and host response. Diabetes Metab. Syndr 14, 407–412 (2020). 32335367 10.1016/j.dsx.2020.04.020 PMC7165108

[21] Lan J Structure of the SARS-CoV-2 spike receptor-binding domain bound to the ACE2 receptor. Nature 581, 215–220 (2020). 32225176 10.1038/s41586-020-2180-5

[22] Cao L De novo design of picomolar SARS-CoV-2 miniprotein inhibitors. Science 370, 426–431 (2020). 32907861 10.1126/science.abd9909 PMC7857403

[23] Algieri C, Donato L & Giorno L Tyrosinase immobilized on a hydrophobic membrane. Biotechnol. Appl. Biochem 64, 92–99 (2017). 26607971 10.1002/bab.1462

[24] Sarma R, Islam MS, Miller A-F & Bhattacharyya D Layer-by-layer-assembled laccase enzyme on stimuli-responsive membranes for chloro-organics degradation. ACS Appl. Mater. Interfaces 9, 14858–14867 (2017). 28397501 10.1021/acsami.7b01999 PMC5787852

[25] Giorno L, Vitola G, Ranieri G & Militano F in Encyclopedia of Membranes (eds Drioli Enrico & Giorno Lidietta) 1–6 (Springer Berlin Heidelberg, 2015).

[26] Sarma R, Islam MS, Running MP & Bhattacharyya D Multienzyme immobilized polymeric membrane reactor for transformation of lignin model compound. LID — 463 [pii] LID - 10.3390/polym10040463 Polymers (2018). 30719335 PMC6358281

[27] Siezen RJ & Leunissen JA Subtilases: the superfamily of subtilisin-like serine proteases. Protein Sci. 6, 501–523 (1997). 9070434 10.1002/pro.5560060301 PMC2143677

[28] Ruan B, London V, Fisher KE, Gallagher DT & Bryan PN Engineering substrate preference in subtilisin: Structural and kinetic analysis of a specificity mutant. Biochemistry 47, 6628–6636 (2008). 18507395 10.1021/bi800089f

[29] Schmitke JL, Stern LJ & Klibanov AM The crystal structure of subtilisin Carlsberg in anhydrous dioxane and its comparison with those in water and acetonitrile. Proc. Natl Acad. Sci 94, 4250 (1997). 9113975 10.1073/pnas.94.9.4250 PMC20708

[30] Wan H Pd/Fe nanoparticle integrated PMAA-PVDF membranes for chloro-organic remediation from synthetic and site groundwater. J. Membr. Sci 594, 117454 (2020). 10.1016/j.memsci.2019.117454 PMC6953629 31929677

[31] Saad A, Mills R, Wan H, Ormsbee L & Bhattacharyya D Thermoresponsive PNIPAm–PMMA-functionalized PVDF membranes with reactive Fe─Pd nanoparticles for PCB degradation. Ind. Eng. Chem. Res 59, 16614–16625 (2020).

[32] Cao P, Mangadlao J & Advincula R Stimuli-responsive polymers and their potential applications in oil-gas industry. Polym. Rev 55, 706–733 (2015).

[33] Rezaei K, Jenab E & Temelli F Effects of water on enzyme performance with an emphasis on the reactions in supercritical fluids. Critical Reviews in Biotechnology 27, 183–195 (2007). 18085461 10.1080/07388550701775901

[34] Ward OP Proteases. Compr. Biotechnol, 604–615, 10.1016/B978-0-444-64046-8.00187-7 (2011).

[35] Xiao S Acute and subchronic toxicities and safety pharmacology studies of a Bacillus Subtilisin in dogs. Biol. Pharm. Bull 44, 211–218 (2021). 33281147 10.1248/bpb.b20-00659

[36] Tian B Construction of pH-responsive and up-conversion luminescent NaYF4:Yb3+/Er3+@SiO_2_@PMAA nanocomposite for colon targeted drug delivery. Sci. Rep 6, 21335 (2016). 26891778 10.1038/srep21335 PMC4759527

[37] Yin Y MSCs-engineered biomimetic PMAA nanomedicines for multiple bioimaging-guided and photothermal-enhanced radiotherapy of NSCLC. J. Nanobiotechnol 19, 80–80 (2021). 10.1186/s12951-021-00823-6 PMC7981797 33743720

[38] Pal P In Groundwater Arsenic Remediation (ed. Pal Parimal) 1–23 (Butterworth-Heinemann, 2015).

[39] Shon HK, Phuntsho S, Chaudhary DS, Vigneswaran S & Cho J Nanofiltration for water and wastewater treatment—a mini review. Drink. Water Eng. Sci 6, 47–53 (2013).

[40] Zhang W-H Graphene oxide membranes with stable porous structure for ultrafast water transport. Nat. Nanotechnol 16, 337–343 (2021). 33479540 10.1038/s41565-020-00833-9

[41] Lee B A carbon nanotube wall membrane for water treatment. Nat. Commun 6, 7109 (2015). 25971895 10.1038/ncomms8109

[42] Zhang G-H High-performance particulate matter including nanoscale particle removal by a self-powered air filter. Nat. Commun 11, 1653 (2020). 32245962 10.1038/s41467-020-15502-7 PMC7125120

[43] Rengasamy S Protection factor for N95 filtering facepiece respirators exposed to laboratory aerosols containing different concentrations of nanoparticles. Ann. Occup. Hyg 59, 373–381 (2015). 25429023 10.1093/annhyg/meu095 PMC4589166

[44] Liang M Efficacy of face mask in preventing respiratory virus transmission: A systematic review and meta-analysis. Travel. Med. Infect. Dis 36, 101751–101751 (2020). 32473312 10.1016/j.tmaid.2020.101751 PMC7253999

[45] Aragaw TA Surgical face masks as a potential source for microplastic pollution in the COVID-19 scenario. Mar. Pollut. Bull 159, 111517–111517 (2020). 32763564 10.1016/j.marpolbul.2020.111517 PMC7381927

[46] Kumar S & Lee HP The perspective of fluid flow behavior of respiratory droplets and aerosols through the facemasks in context of SARS-CoV-2. Phys. Fluids 32, 111301–111301 (2020). 10.1063/5.0029767 PMC7713871 33281434

[47] Nagy E In Basic Equations of Mass Transport Through a Membrane Layer (Second Edition) (ed. Nagy Endre) 21–68 (Elsevier, 2019).

[48] Mandal G, Kumar A, Sharma DC & Kumar H Comparative analysis of different air density equations. MAPAN 28, 51–62 (2013).

[49] Zohar Y, Lee SYK, Lee WY, Jiang L & Tong PIN Subsonic gas flow in a straight and uniform microchannel. J. Fluid Mech 472, 125–151 (2002).

[50] Hwang S-T & Kammermeyer K In Permeability of Plastic Films and Coatings: To Gases, Vapors, and Liquids (ed. Hopfenberg Harold B.) 197–205 (Springer US, 1974).

[51] Iversen SB, Bhatia VK, Dam-Johansen K & Jonsson G Characterization of microporous membranes for use in membrane contactors. J. Membr. Sci 130, 205–217 (1997).

[52] Lesimple A, Jasim SY, Johnson DJ & Hilal N The role of wastewater treatment plants as tools for SARS-CoV-2 early detection and removal. J. Water Process Eng 38, 101544–101544 (2020). 10.1016/j.jwpe.2020.101544 PMC7377730 38620686

[53] Huang H-L & Yang S Filtration characteristics of polysulfone membrane filters. J. Aerosol Sci 37, 1198–1208 (2006).

[54] Burton NC, Grinshpun SA & Reponen T Physical collection efficiency of filter materials for bacteria and viruses. Ann. Occup. Hygiene 51, 143–151 (2007). 10.1093/annhyg/mel073 PMC7187796 17041245

[55] Lausted CG Maximum static inspiratory and expiratory pressures with different lung volumes. Biomed. Eng. Online 5, 29–29 (2006). 16677384 10.1186/1475-925X-5-29 PMC1501025

[56] Stewart M & Arnold K In Gas-Liquid And Liquid-Liquid Separators (eds Stewart Maurice & Arnold Ken) 65–130 (Gulf Professional Publishing, 2008).

[57] Sant’Ana AS In Encyclopedia of Food Microbiology (Second Edition) (eds. Batt Carl A. & Tortorello Mary Lou) 36–41 (Academic Press, 2014).

[58] Dbouk T & Drikakis D On respiratory droplets and face masks. Phys. Fluids 32, 063303–063303 (2020). 10.1063/5.0015044 PMC7301882 32574231

[59] Leung NHL Respiratory virus shedding in exhaled breath and efficacy of face masks. Nat. Med 26, 676–680 (2020). 32371934 10.1038/s41591-020-0843-2 PMC8238571

[60] Asadi S Efficacy of masks and face coverings in controlling outward aerosol particle emission from expiratory activities. Sci. Rep 10, 15665 (2020). 32973285 10.1038/s41598-020-72798-7 PMC7518250

[61] Yang W, Elankumaran S & Marr LC Concentrations and size distributions of airborne influenza A viruses measured indoors at a health centre, a day-care centre and on aeroplanes. J. R. Soc. Interface 8, 1176–1184 (2011). 21300628 10.1098/rsif.2010.0686 PMC3119883

[62] Rengasamy S, Miller A & Eimer BC Evaluation of the filtration performance of NIOSH-approved N95 filtering facepiece respirators by photometric and number-based test methods. J. Occup. Environ. Hygiene 8, 23–30 (2011). 10.1080/15459624.2010.515556 21154105

[63] Bae S Effectiveness of surgical and cotton masks in blocking SARS–CoV-2: A controlled comparison in 4 patients. Ann. Intern. Med 173, W22–W23 (2020). 32251511 10.7326/M20-1342 PMC7153751

[64] Rampado R, Crotti S, Caliceti P, Pucciarelli S & Agostini M Recent advances in understanding the protein corona of nanoparticles and in the formulation of “Stealthy” nanomaterials. Front. Bioeng. Biotechnol 8, 10.3389/fbioe.2020.00166 (2020). PMC7145938 32309278

[65] Guerrini L, Alvarez-Puebla RA & Pazos-Perez N Surface modifications of nanoparticles for stability in biological fluids. Materials 11, 10.3390/ma11071154 (2018). PMC6073273 29986436

[66] Gupta JK, Lin Ch Fau-Chen Q & Chen Q Characterizing exhaled airflow from breathing and talking. Indoor Air 20, 31–39 (2010). 20028433 10.1111/j.1600-0668.2009.00623.x

[67] Satarker S & Nampoothiri M Structural proteins in severe acute respiratory Syndrome Coronavirus-2. Arch. Med. Res 51, 482–491 (2020). 32493627 10.1016/j.arcmed.2020.05.012 PMC7247499

[68] Viswanath S, Wang J, Bachas LG, Butterfield DA & Bhattacharyya D Site-directed and random immobilization of subtilisin on functionalized membranes: Activity determination in aqueous and organic media. Biotechnol. Bioeng 60, 608–616 (1998). 10099469

[69] Vishwanath S, Bhattacharyya D, Huang W & Bachas LG Site-directed and random enzyme immobilization on functionalized membranes: kinetic studies and models. J. Membr. Sci 108, 1–13 (1995).

[70] Cen Y-K, Liu Y-X, Xue Y-P & Zheng Y-G Immobilization of enzymes in/on membranes and their applications. Adv. Synth. Catal 361, 5500–5515 (2019).

[71] Chen P-C, Ma Z, Zhu X-Y, Chen D-J & Huang X-J Fabrication and optimization of a lipase immobilized enzymatic membrane bioreactor based on polysulfone gradient-pore hollow fiber membrane. Catalysts 9, 10.3390/catal9060495 (2019).

[72] Sun XS In Bio-Based Polymers and Composites (eds. Wool Richard P. & Sun Xiuzhi Susan) 292–326 (Academic Press, 2005).

[73] Durowoju IB, Bhandal KS, Hu J, Carpick B & Kirkitadze M Differential Scanning Calorimetry–A method for assessing the thermal stability and conformation of protein antigen. J Vis Exp., 55262, 10.3791/55262 (2017). PMC5409303 28287565

[74] Mazurenko S Exploration of protein unfolding by modelling calorimetry data from reheating. Sci. Rep 7, 16321 (2017). 29176711 10.1038/s41598-017-16360-y PMC5701188

[75] Konieczny P DSC and electrophoretic studies on protein denaturation of Anodonta woodiana (Lea, 1834). J. Therm. Anal. Calorim 126, 69–75 (2016).

[76] Giancola C, De Sena C, Fessas D, Graziano G & Barone G DSC studies on bovine serum albumin denaturation Effects of ionic strength and SDS concentration. Int. J. Biol. Macromol 20, 193–204 (1997). 9218168 10.1016/s0141-8130(97)01159-8

[77] Huynh K & Partch CL Analysis of protein stability and ligand interactions by thermal shift assay. Curr. Protoc. Protein Sci 79, 28.29.21–28.29.14, 10.1002/0471140864.ps2809s79 (2015). 25640896 PMC4332540

[78] Biggar KK, Dawson NJ & Storey KB Real-time protein unfolding: a method for determining the kinetics of native protein denaturation using a quantitative real-time thermocycler. BioTech. 53, 231–238 (2012). 10.2144/0000113922 23046506

[79] Vedadi M Chemical screening methods to identify ligands that promote protein stability, protein crystallization, and structure determination. Proc. Natl Acad. Sci 103, 15835 (2006). 17035505 10.1073/pnas.0605224103 PMC1595307

[80] Vladisavljević GT In Nanoemulsions (eds. Jafari Seid Mahdi & McClements David Julian) 287–346 (Academic Press, 2018).

[81] Xue X, Ball JK, Alexander C & Alexander MR All surfaces are not equal in contact transmission of SARS-CoV-2. Matter 3, 1433–1441 (2020). 33043292 10.1016/j.matt.2020.10.006 PMC7538118

[82] Wang L-Y, Yu LE & Chung T-S Effects of relative humidity, particle hygroscopicity, and filter hydrophilicity on filtration performance of hollow fiber air filters. J. Membr. Sci 595, 117561 (2020).

[83] Bhardwaj R & Agrawal A Tailoring surface wettability to reduce chances of infection of COVID-19 by a respiratory droplet and to improve the effectiveness of personal protection equipment. Phys. Fluids 32, 081702 (2020). 10.1063/5.0020249 PMC8728633 35002197

[84] Bhardwaj R & Agrawal A Likelihood of survival of coronavirus in a respiratory droplet deposited on a solid surface. Phys. Fluids 32, 061704 (2020). 10.1063/5.0012009 PMC7295365 32574230

[85] Badawy J, Nguyen OK, Clark C, Halm EA & Makam AN Is everyone really breathing 20 times a minute? Assessing epidemiology and variation in recorded respiratory rate in hospitalised adults. BMJ Qual. Saf 26, 832–836 (2017). 10.1136/bmjqs-2017-006671 PMC5812442 28652259

[86] Patrício Silva AL Increased plastic pollution due to COVID-19 pandemic: Challenges and recommendations. Chem. Eng. J 405, 126683–126683 (2021). 32834764 10.1016/j.cej.2020.126683 PMC7430241

[87] Tabka Z, Jebria AB & Guénard H Effect of breathing dry warm air on respiratory water loss at rest and during exercise. Respir. Physiol 67, 115–125 (1987). 3823651 10.1016/0034-5687(87)90034-x

[88] Islam MS, Hernández S, Wan H, Ormsbee L & Bhattacharyya D Role of membrane pore polymerization conditions for pH responsive behavior, catalytic metal nanoparticle synthesis, and PCB degradation. J. Membr. Sci 555, 348–361 (2018). 10.1016/j.memsci.2018.03.060 PMC6358284 30718939

[89] Baldridge KC Demonstration of Hollow fiber membrane-based enclosed space air remediation for capture of an aerosolized synthetic SARS-CoV-2 mimic and pseudovirus particles. ACS ES&T Eng. 2, 251–262 (2022). 10.1021/acsestengg.1c00369 PMC8768008 37406036

[90] Stadlbauer D SARS-CoV-2 Seroconversion in humans: A detailed protocol for a serological assay, antigen production, and test setup. Curr. Protoc. Microbiol 57, e100 (2020). 32302069 10.1002/cpmc.100 PMC7235504

