# Supplementary material for: Aerosol capture and coronavirus spike protein deactivation by enzyme functionalized antiviral membranes
Source: Commun Mater. Author manuscript; Available in PMC 2022 Nov 18. (PMC9674191; doi:10.1038/s43246-022-00256-0)
Supplement: Supplementary Material [file NIHMS1822857-supplement-Supplementary_Material.pdf]

Supplementary Materials for

Aerosol capture and coronavirus spike protein deactivation by enzyme functionalized antiviral membranes

Authors: Rollie Mills[1], Ronald J. Vogler[1] †, Matthew Bernard[1] †, Jacob Concolino[1], Louis B. Hersh[2], Yinan Wei[3], Jeffrey Todd Hastings[4], Thomas Dziubla[1], Kevin C. Baldridge[1] and Dibakar Bhattacharyya[1*]

Affiliations:

[1]Department of Chemical and Materials Engineering, University of Kentucky; Lexington, KY 40506, USA

[2]Department of Molecular and Cellular Biochemistry, University of Kentucky; Lexington, KY 40506, USA

[3]Department of Chemistry, University of Kentucky; Lexington, KY 40506, USA

[4]Department of Electrical and Computer Engineering, University of Kentucky; Lexington, KY 40506, USA

† These authors contributed equally to this work.

***Corresponding author email: db@uky.edu**

This PDF file includes:

Supplementary Figure 1 to 17

Supplementary Table 1 to 6

Supplementary References

Supplementary Text

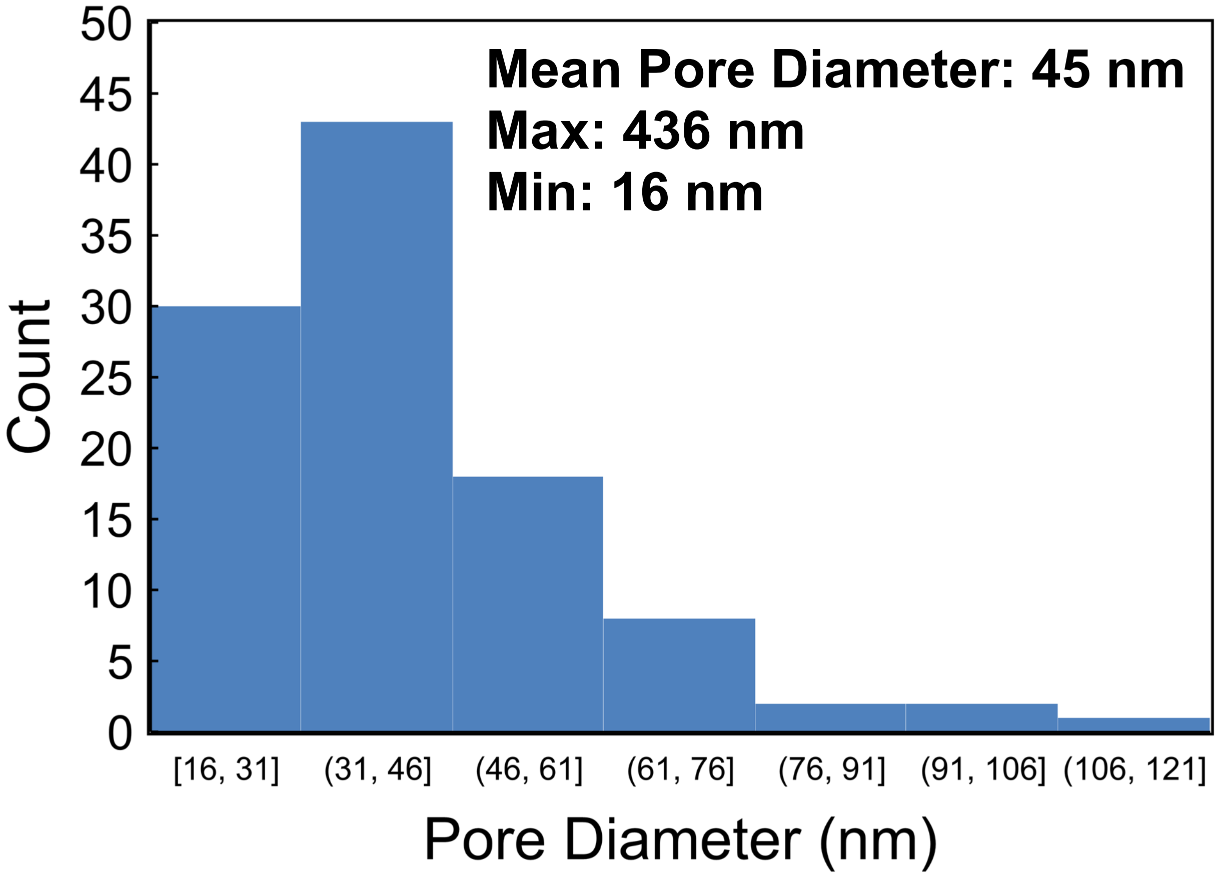

Supplementary Figure. 1. Pore diameter distribution of PVDF400. Measured from three different sample images using ImageJ and SEM surface images.

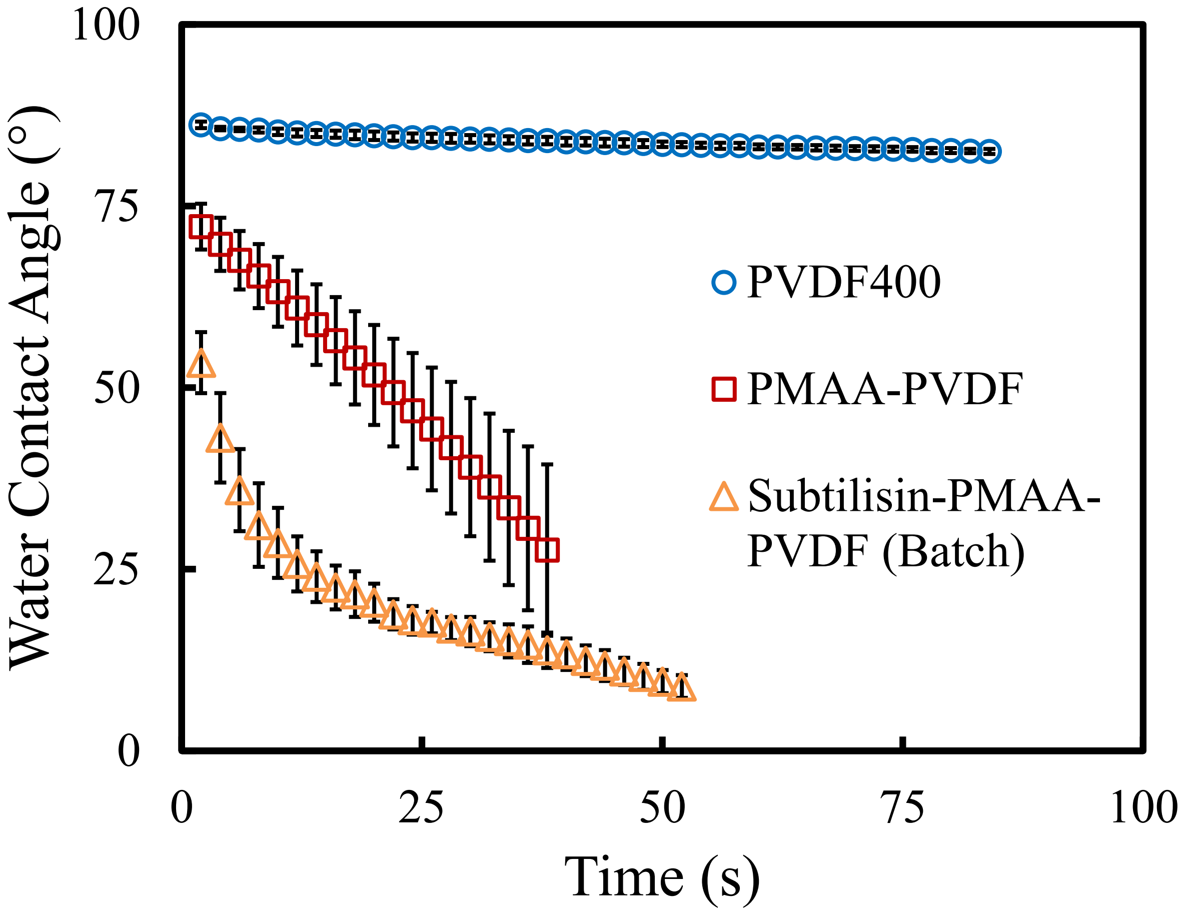

Supplementary Figure 2. The water contact-angle of PVDF400, PMAA-PVDF, and subtilisin-PMAA-PVDF (batch mode) membranes as a function of time. The sessile-drop method was used to measure contact-angle and the water pH was ~6. The PMAA-PVDF membranes had weight gain of 3.3% with PMAA functionalization. The subtilisin-PMAA-PVDF membranes had ~0.07 mg enzymes immobilized/cm^2 of membrane. Error bars represent the standard deviation of 3 measurements taken at different locations on the samples. Blue circles were used for PVDF400, red squares were used for PMAA-PVDF, and yellow triangles were used for Subtilisin-PMAA-PVDF (batch).

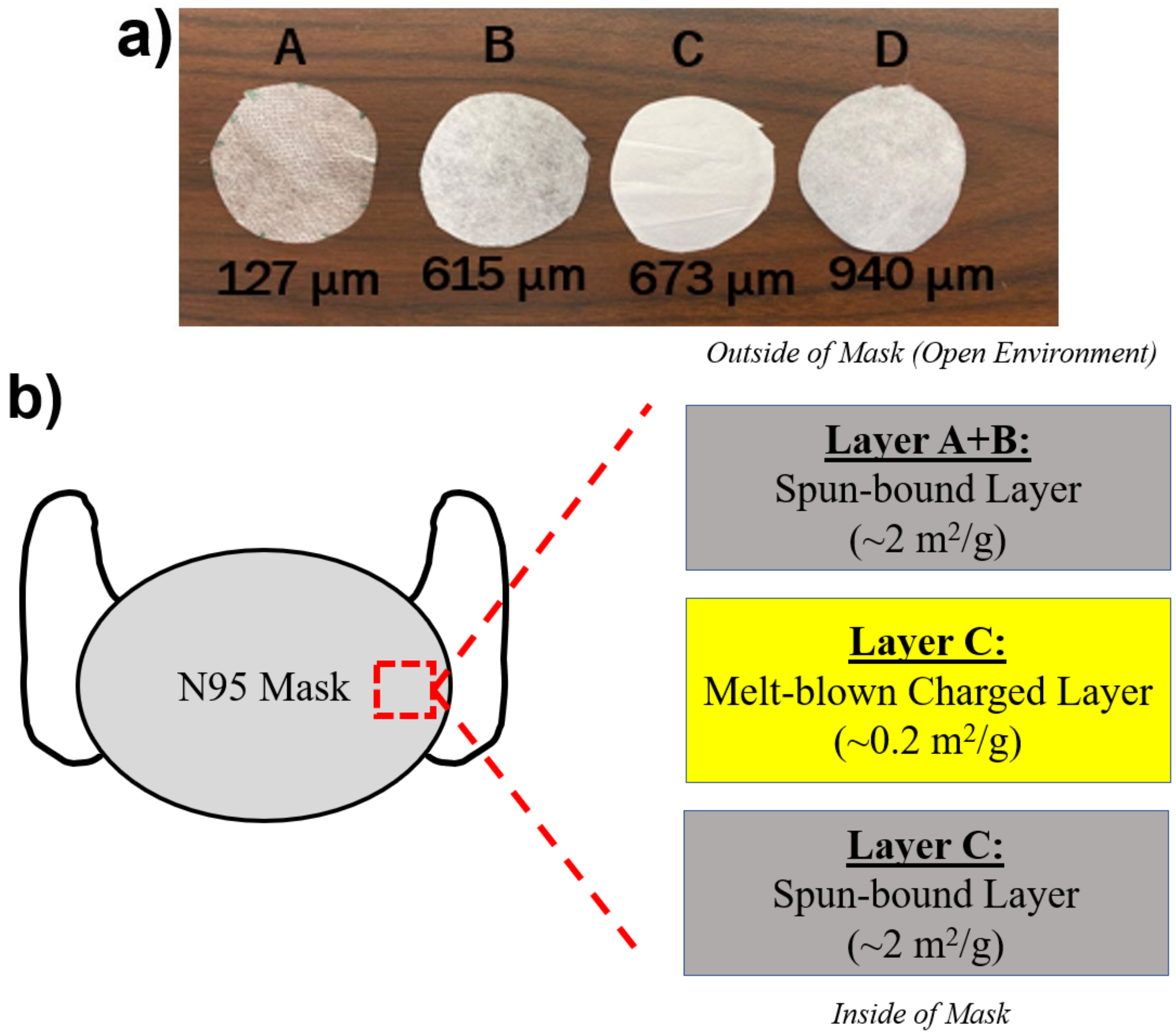

Supplementary Figure 3. Structure of each layer for commercial N95 mask. (a) Images of separated N95 mask layers and measured thicknesses (measured from triplicate samples). (**b**) Schematic depicting orientation of layers of N95 mask, along with type of fibrous filter. In mask orientation, Layer A is exposed to the open environment and Layer D is exposed to the individual. Layer C is the separating charged layer.

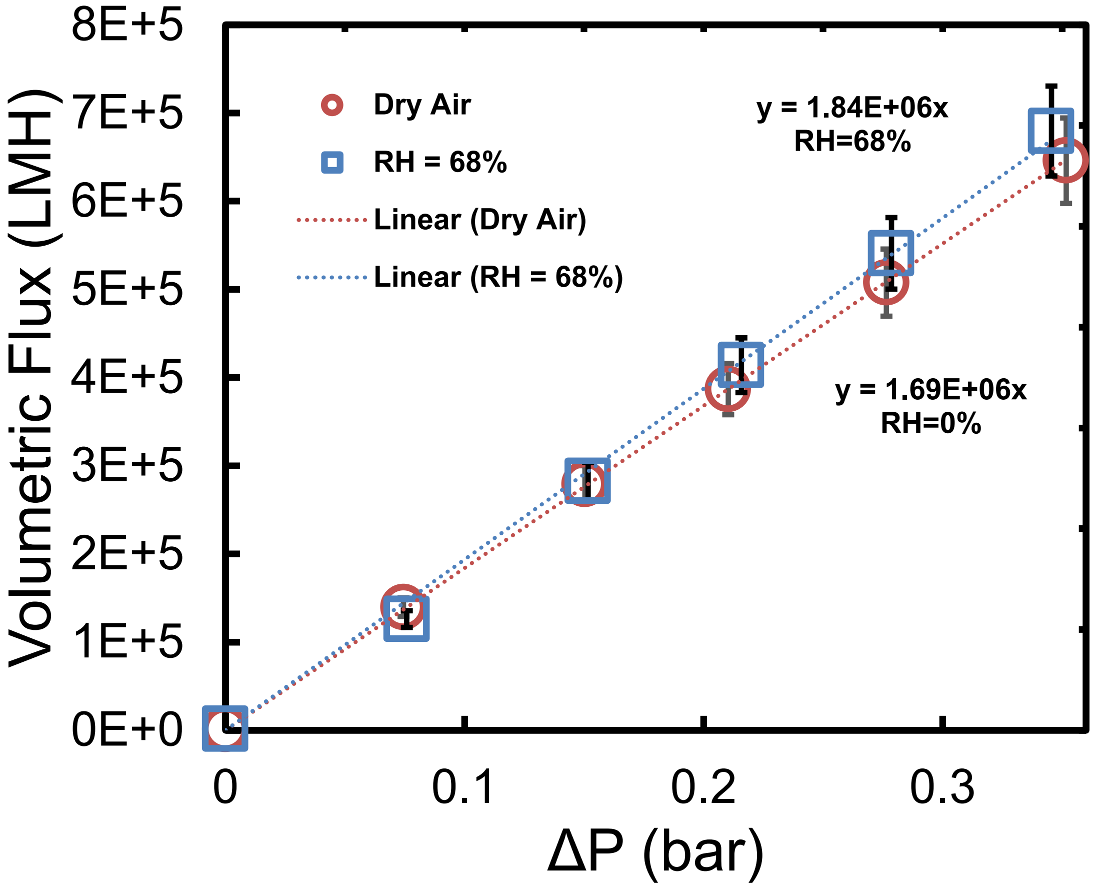

Supplementary Figure 4. Effect of relative humidity (RH) on membrane air permeability (LMH or liters/m^2/hour for volumetric, kg/m^2/hour for mass) of PMAA-PVDF membranes. Dry-air was introduced from an ultra-pure dry-air tank and air with relative humidity of 68% was produced using a bubbler. Error bars represent the standard deviation of 3 different measurements taken on the samples. Measured flow rate measurements normalized at STP. Dry air flow is displayed with red circles and dashed line. RH=68% flow is displayed with blue squares and dashed line.

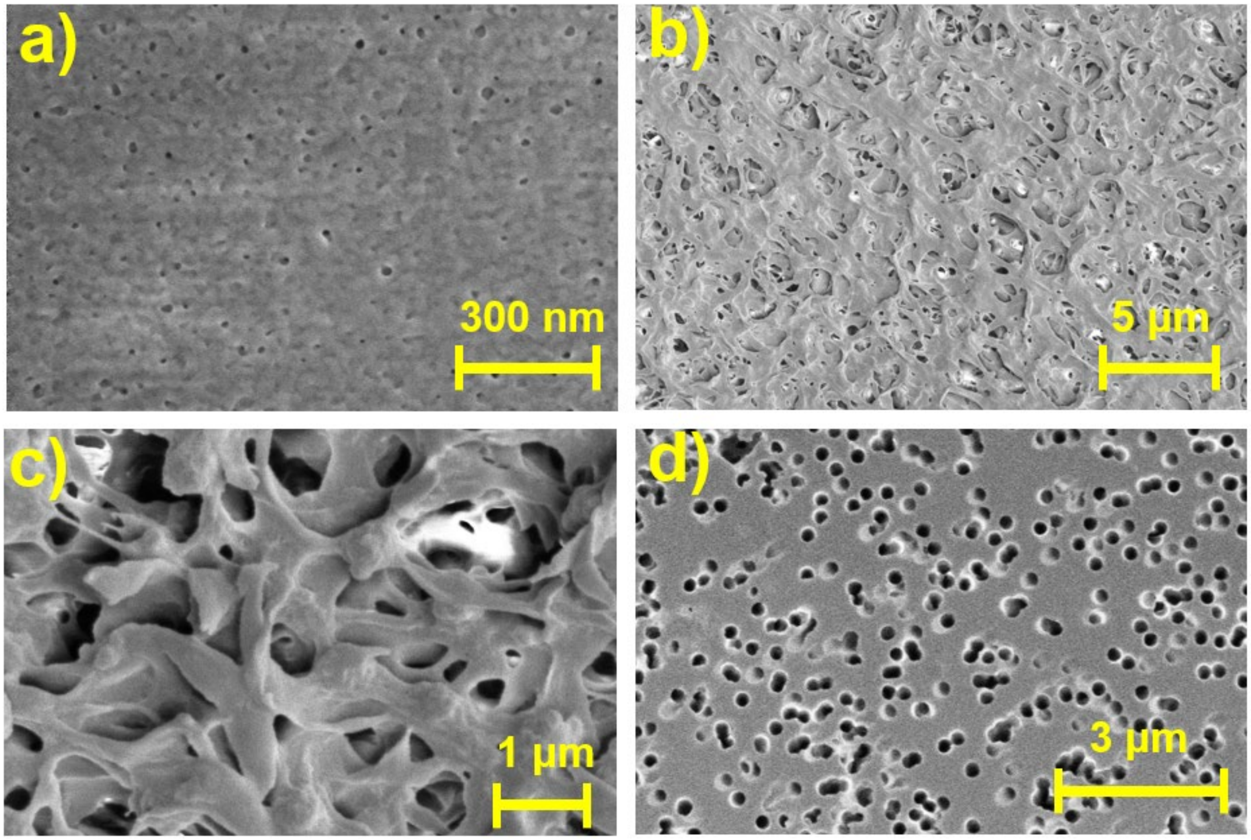

Supplementary Figure 5. SEM imaging of MF membranes utilized for air/water flow characterization. Surface image of (**a**) PS35, (**b**) Hydrophilic Durapore (100 nm pore diameter), (**c**) Hydrophilic Durapore (220 nm pore diameter), and (**d**) Isopore (200 nm pore diameter).

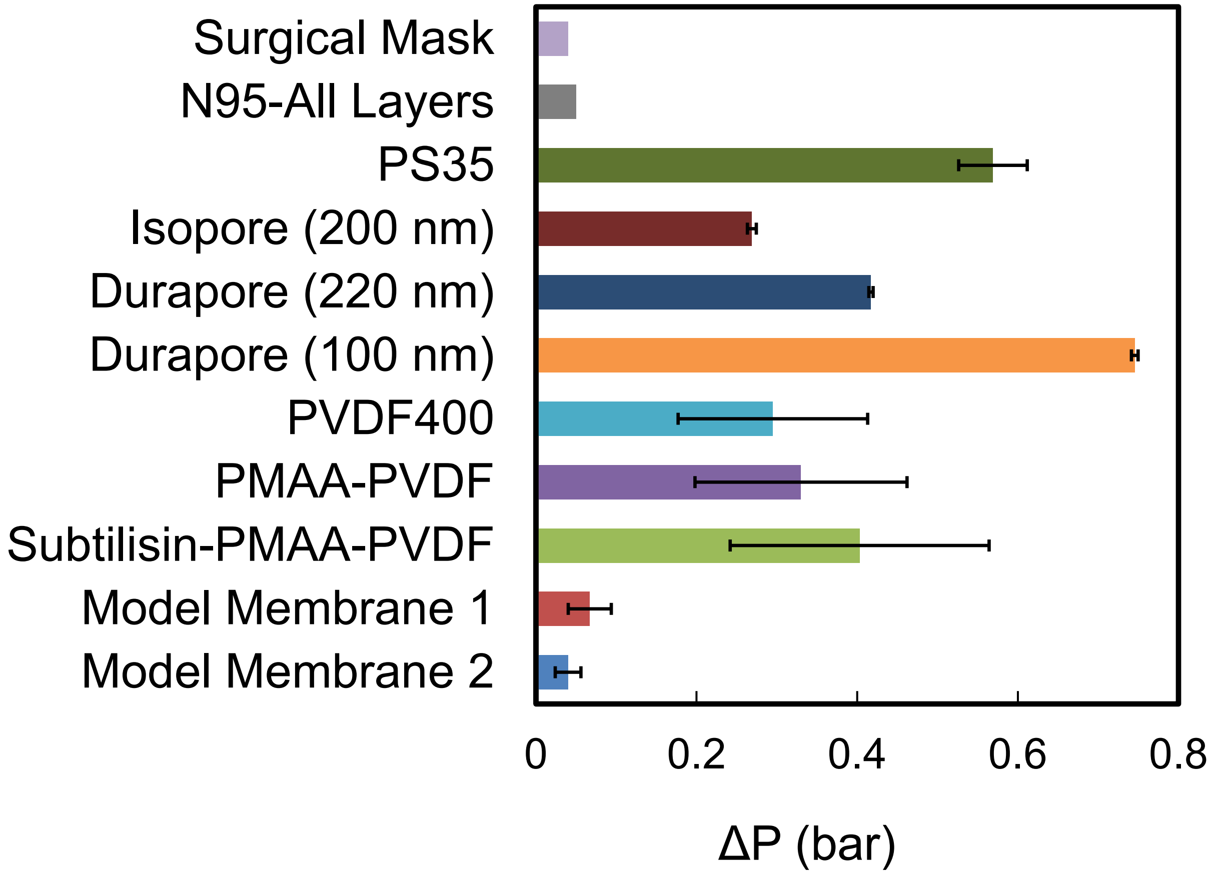

Supplementary Figure 6. Pressure drop across different membrane and mask material at a consistent airflow rate of 7.5 L/min of dry-air over 9.32 cm^2 of membrane and mask area. Error bars represent the standard deviation of 3 different measurements taken on the samples. Surgical mask is displayed as a light purple bar, N95-All Layers is displayed as a gray bar, PS35 is displayed as a dark green bar, Isopore-200 nm is displayed as a dark red bar, Durapore-220 nm is displayed as a dark blue bar, Durapore-100 nm is displayed as an orange bar, PVDF400 is displayed as a light blue bar, PMAA-PVDF is displayed as a dark purple bar, Subtilisin-PMAA-PVDF is displayed as a light green bar, Model Membrane 1 is displayed as a light red bar, and Model Membrane 2 is displayed as a blue bar.

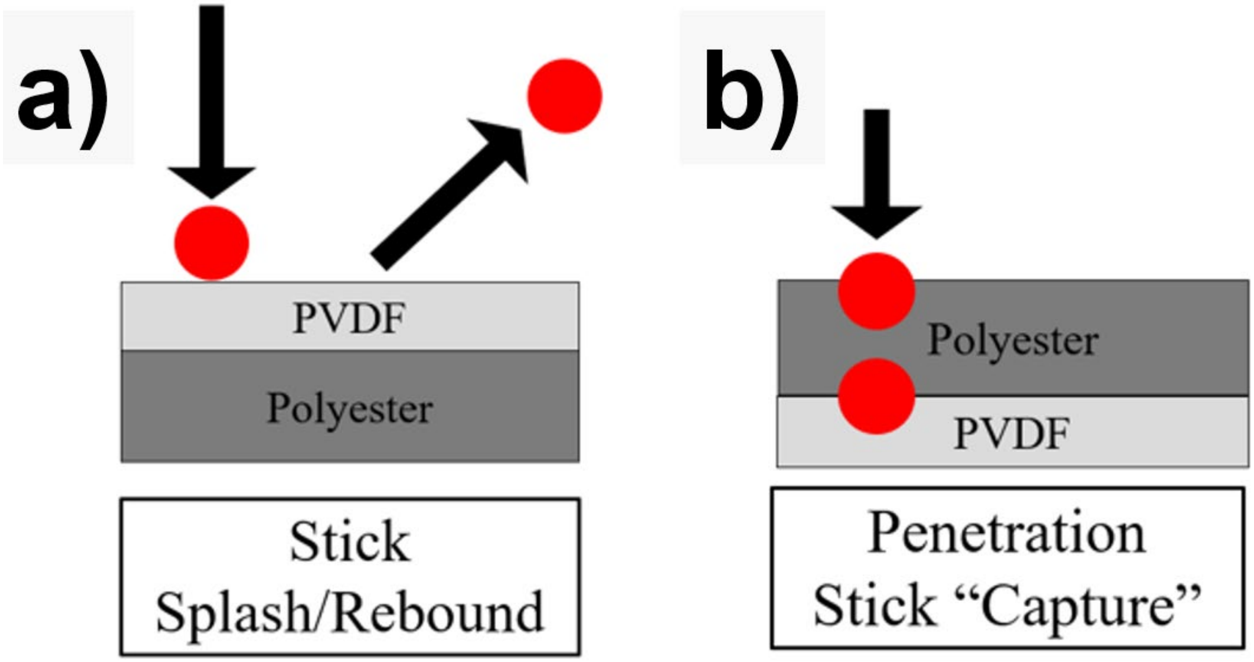

Supplementary Figure 7. Schematic of dual-mode particle capture by PVDF400 membrane. (**a**) Normal and (**b**) reverse orientation of aerosol particle capture by PVDF400. Normal and reverse orientation consists of the PVDF and polyester layer, respectively, being exposed to the feed inlet side.

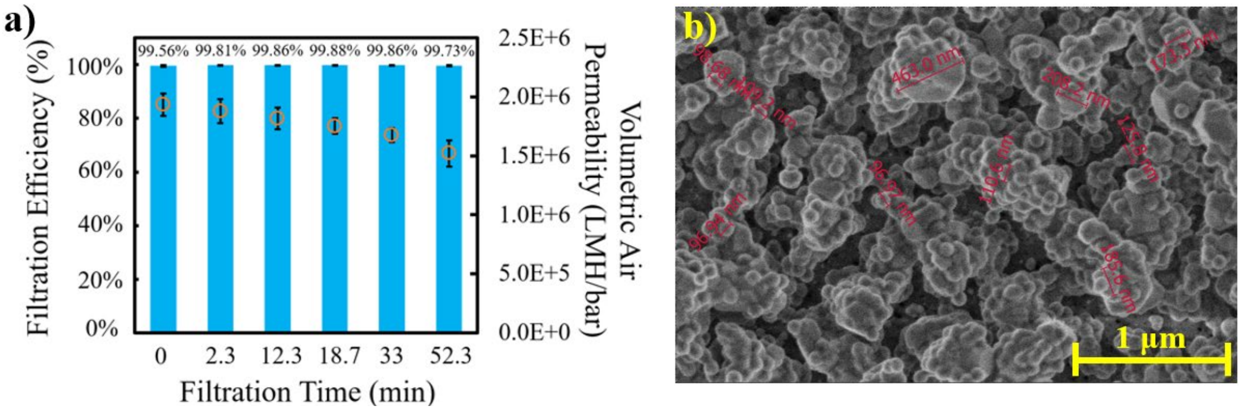

Supplementary Figure 8. Effect of particle surface proteins on membrane filtration capabilities. (a) Filtration efficiency of PSL-sfGFP aerosols using PVDF400 flat sheet membranes at a relative humidity of 16-22% with measured membrane permeability drop (LMH or liters per m^2 per hour) as a function of filtration time. Average concentration of feed air was ~19,000 particles/L of 0.3-0.5 μm sized PSL aerosols. Error bars represent standard deviation of triplicate calculated permeability values. Particle count measured using Met One Instruments' GT-526S particle counter. Flow rate measurements normalized at STP. Error bars represent the standard deviation of 3 different measurements taken on the samples. Blue bars represent filtration efficiency and orange circles represent volumetric air permeability. **(b)** SEM image of PVDF400 membrane after PSL-sfGFP (with protein) aerosol filtration for ~50 minutes.

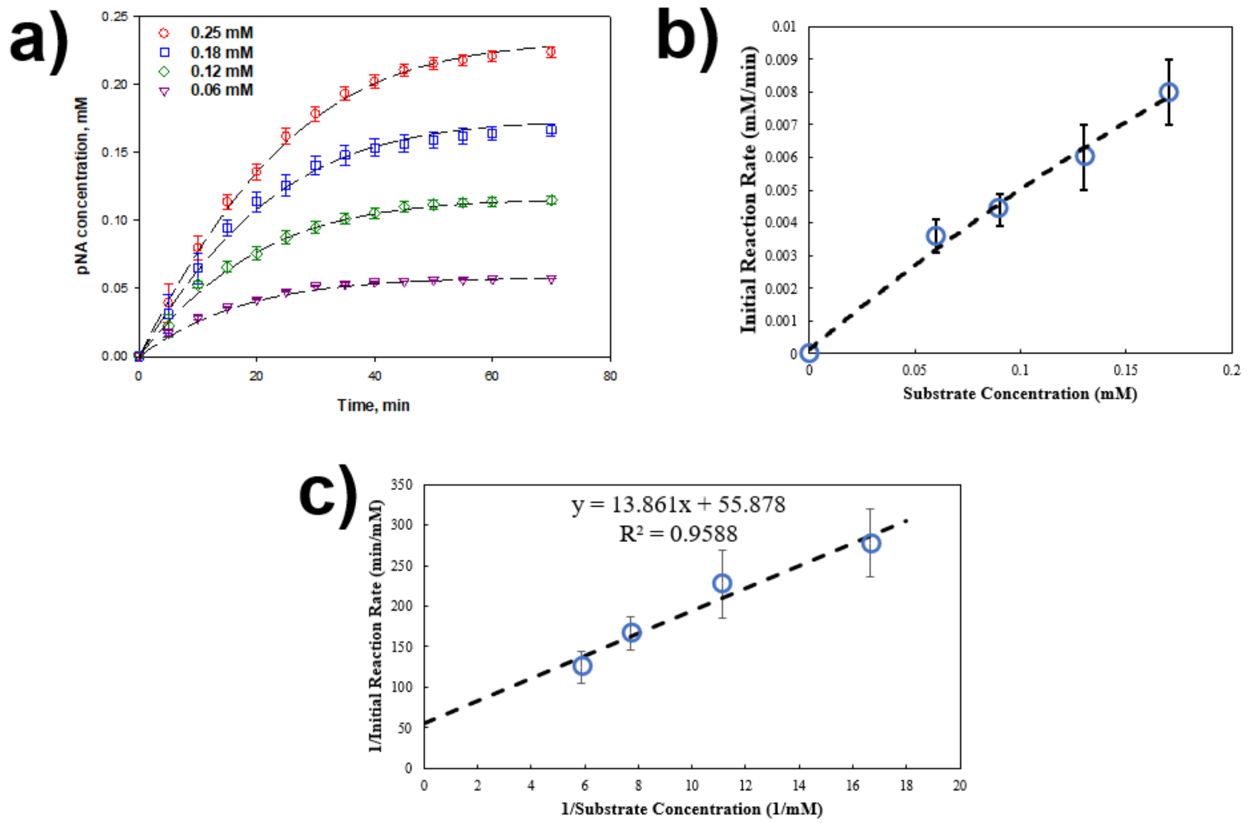

Supplementary Figure 9. Activity characterization of subtilisin Carlsberg in solution. (a) Polypeptide (N-succinyl-Ala-Ala-Pro-Phe-p-nitroanilide) proteolysis versus time. Red circles, blue squares, green diamonds, and purple reverse pyramids represent 0.25, 0.18, 0.12, and 0.06 mM reactant concentration. (**b**) Michaelis-Menten plot (low substrate concentrations), represented as blue circles and (**c**) Lineweaver-Burk plot catalyzed by subtilisin Carlsberg, represented with blue circles. Error bars represent the standard deviation of triplicate experimentation. The substrate concentration ranged from 0.06-0.25 mM at pH 8 at 37 °C. 0.12 mg/L subtilisin was added initially. pH was buffered by an addition of 1.5 g/L $NaHCO_3$. K_M and V_{max} were found to be 0.25 mM and 0.15 mmol/mg·min.

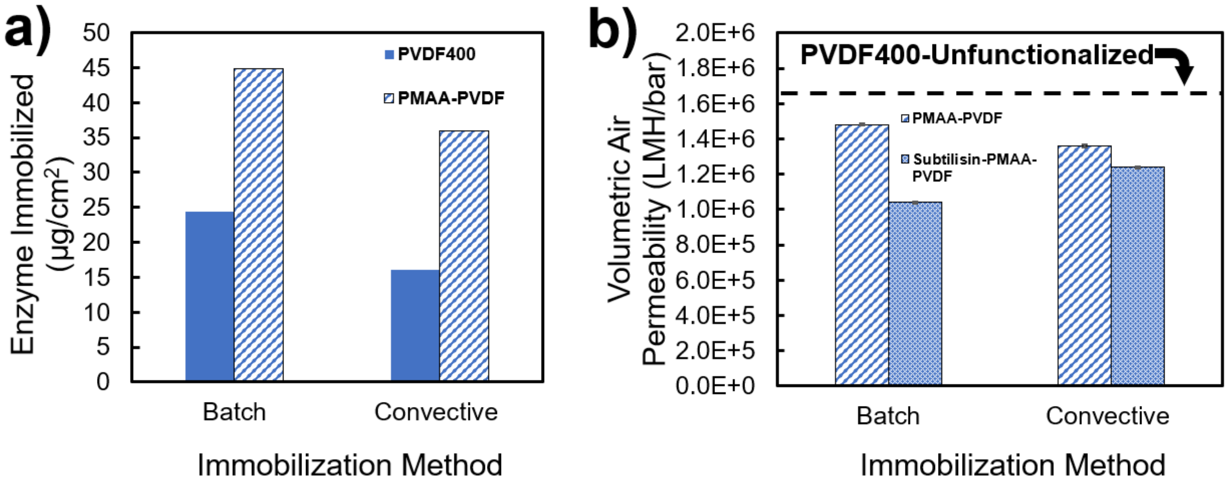

Supplementary Figure 10. Effect of polymer functionalization on mass of enzyme immobilized and effect of polymer/enzyme functionalization on membrane permeability. (a) Subtilisin Carlsberg functionalization of PMAA-PVDF (single sample, 2% weight gain) and unfunctionalized PVDF400 membranes with batch and convective mode. Membrane area was 9.32 cm^2 and 100 mL of 0.1 mg/mL enzyme solution was used for immobilization process. **(b)** Air permeability decrease of PVDF400 membrane after PMAA (1.5-3%) and subtilisin functionalization (Subtilisin-PMAA-PVDF) with batch and convective immobilization method. Flow rate measurements normalized at STP. PVDF flows are at normal orientation. Error bars represent the standard deviation of 3 different measurements taken on the samples.

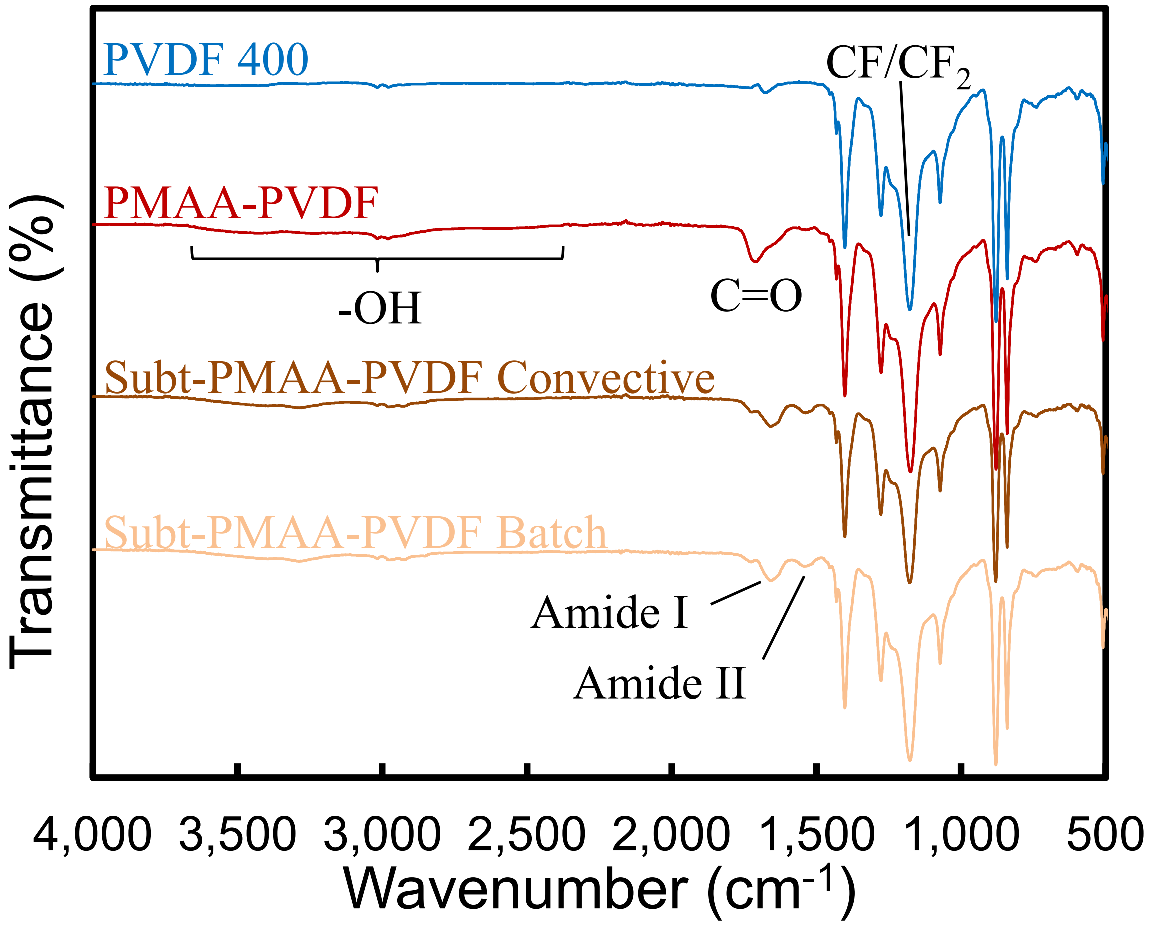

Supplementary Figure 11. FTIR spectra of a PVDF 400 membrane, a PMAA-PVDF membrane, a Subtilisin-PMAA-PVDF that underwent batch enzyme functionalization, and a Subtilisin-PMAA-PVDF membrane that underwent convective functionalization. All functionalized membranes had a PMAA weight gain of 3.4%. Subtilisin-PMAA-PVDF samples were analyzed after the flow of dry-air through the membrane. All spectra are an average of 32 scans with a resolution of 4 cm^{-1}. Spectral data was collected with a Nicolet™ iS50 FTIR Spectrometer (Thermo Scientific™) at a resolution of 4 cm^{-1}; reported spectra were averages of 32 scans. Subtilisin-PMAA-PVDF membranes were analyzed after the filtration of ultra-pure dry-air. All membranes showed the CF/CF_2 peak at ~1,175 cm^{-1} corresponding to PVDF 400 base membrane [1]. As expected, the PMAA-PVDF membrane showed the broad -OH peak (~3600 cm^{-1} to 2400 cm^{-1}) and the C=O (~1,710 cm^{-1}) corresponding to the carboxylic acid groups of PMAA [1]. The Amide I and Amide II peaks on the spectra for the Subt-PMAA-PVDF samples at ~1,655 cm^{-1} and ~1,540 cm^{-1}, respectively, verified the incorporation of enzymes in batch and convective mode [2-4].

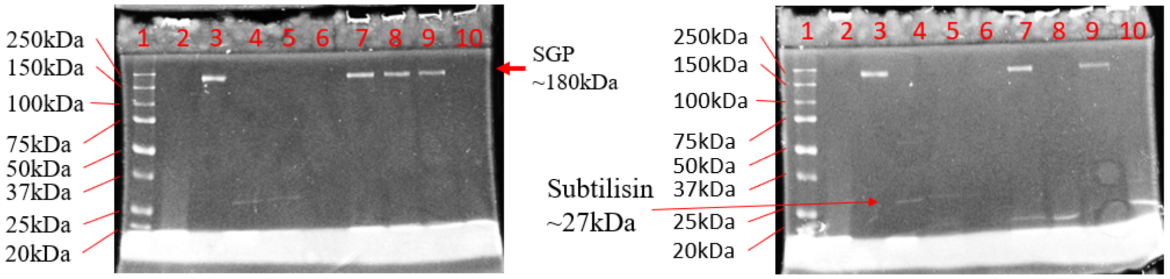

Trial 1

1. BioRad Allblue Precision Plus protein marker
2. PSL only (15uL)
3. SGP only (15uL)
4. Subtilisin only (15uL)
5. SGP + subtilisin-PMAA-PVDF400 (30uL)
6. PBS + subtilisin-PMAA-PVDF400 (30uL)
7. Untreated SGP-PSL (30uL)
8. SGP-PSL + unfunctionalized PVDF400 (30uL)
9. SGP-PSL + PMAA-PVDF400 (30uL)
10. SGP-PSL + subtilisin-PMAA-PVDF400 (30uL)

Trial 2

1. BioRad Allblue Precision Plus protein marker
2. PSL only (15uL)
3. SGP only (15uL)
4. Subtilisin only (15uL)
5. SGP + subtilisin-PMAA-PVDF400 (30uL)
6. PBS + subtilisin-PMAA-PVDF400 (30uL)
7. Untreated SGP-PSL (30uL)
8. SGP-PSL + subtilisin-PMAA-PVDF400 (60 s reaction time) (30uL)
9. SGP-PSL + PMAA-PVDF400 (30uL)
10. SGP-PSL + subtilisin-PMAA-PVDF400 (30uL)

Supplementary Figure 12. SDS-PAGE analysis of 100 nm SGP-functionalized PSL after 30 second reaction (unless stated otherwise) with different membrane surfaces. Approximately 2.6 µg of protein was loaded in each well (except the SGP-only lane with ~5.2 µg) with reactions carried out at total protein concentrations of ~87.5 µg/mL at pH of 7.8 and 23°C. These experiments were performed in duplicate.

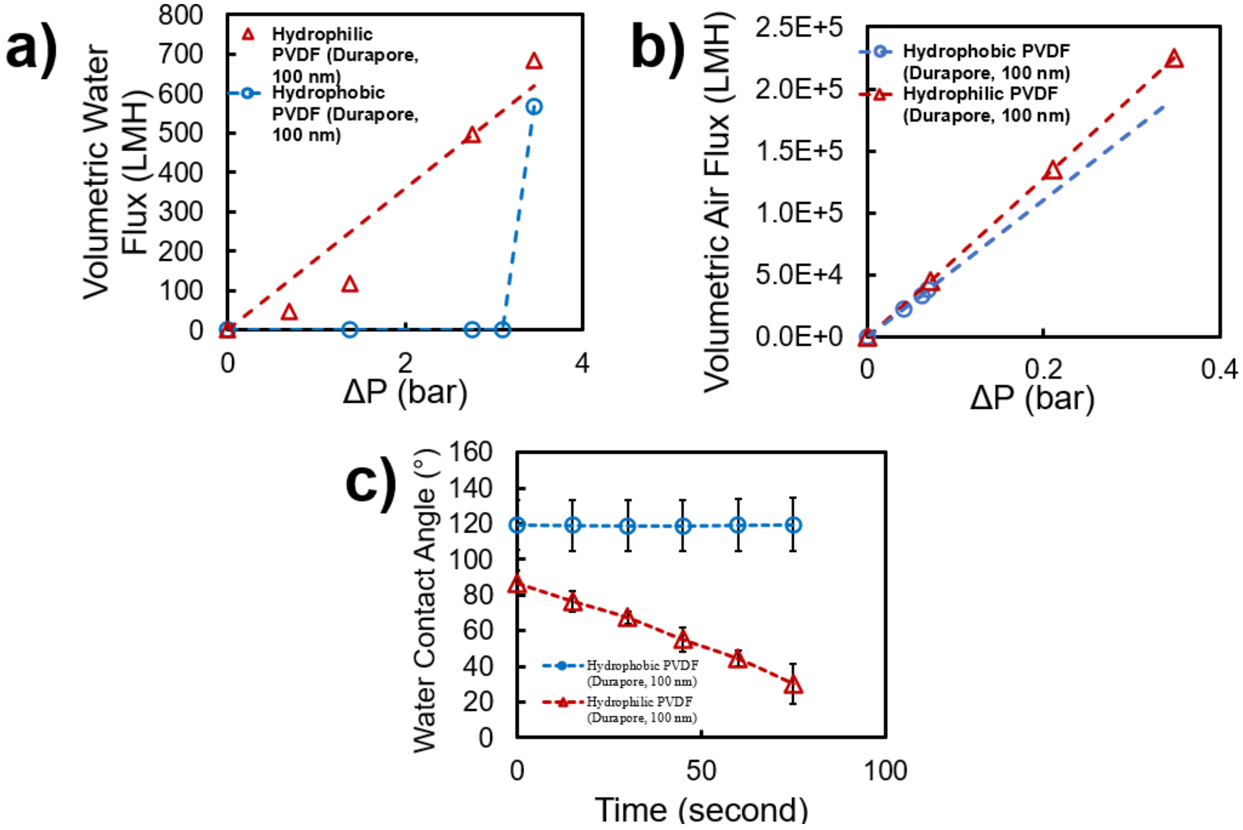

Supplementary Figure 13. Flow characterization of hydrophobic versus hydrophilic membranes. (**a**) Volumetric water flux (LMH, liter per m^2 per h)) and (**b**) volumetric air flux (LMH at 0% RH) of hydrophobic and hydrophilic PVDF Durapore membranes (average pore diameter: 100 nm). Single sample measurements were taken. (**c**) Water contact-angle of hydrophobic and hydrophilic PVDF Durapore membranes (average pore diameter: 100 nm) as a function of time. The sessile-drop method was used to measure contact-angle and the water pH was ~6. Error bars represent the standard deviation of 3 measurements taken at different locations on the samples. Note in (a) minimum water entry pressure required for hydrophobic membrane water flow as expected by Young-Laplace equation relating contact angle and pore diameter. Error bars represent the standard deviation of 3 different measurements taken on the samples. Blue circles and dashed lines were used for the hydrophobic membrane. Red triangles and dashed liens were used for the hydrophilic membrane.

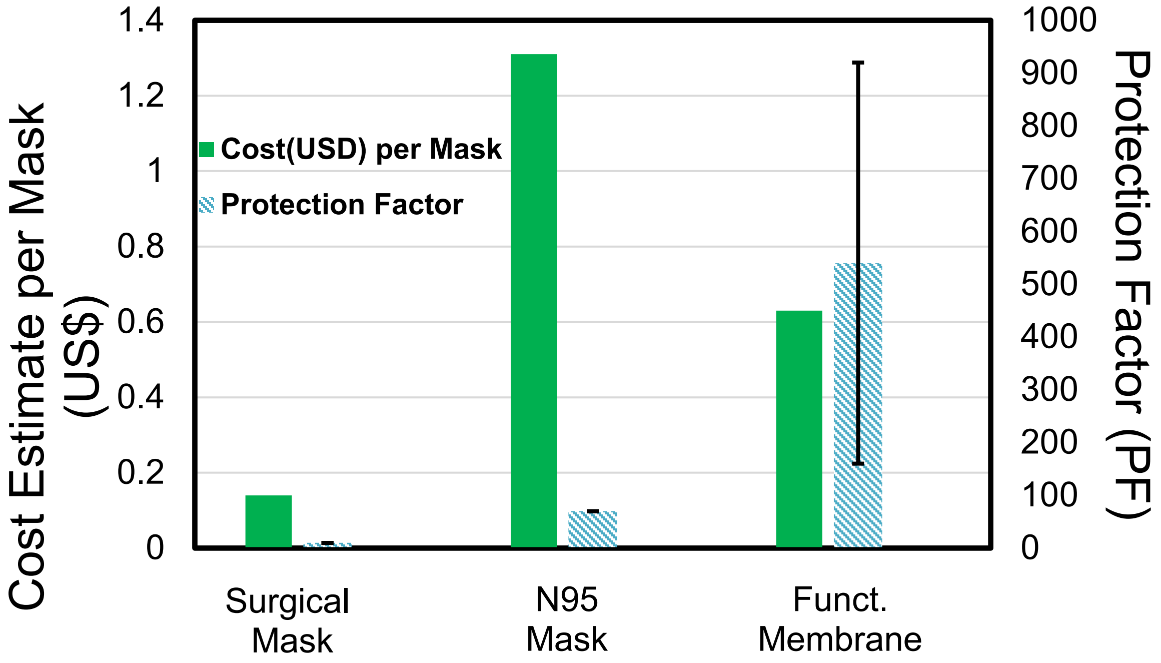

Supplementary Figure 14. Cost analysis (US$) with corresponding protection factors (for coronavirus-sized particles) of different mask material. Cost of functionalized membrane estimated from cost of individual materials (from Sigma Aldrich) for 230 cm^2 (common area of respiratory face mask) with PMAA weight gain of 3% and enzyme batch immobilization of 45 µg/ cm^2. Protection factor for N95 and surgical are high-end values found in literature [5,6]. Note that the cost for surgical and N95 masks are commercial purchase costs obtained from literature [7] and 3M's website (valid prices as of September 16, 2021). Error bars of functionalized membrane represent the standard deviation of 3 different measurements taken on the samples. Error bars for protection factor of surgical and N95 mask represent standard deviation reported in literature [6]. Green bars are used for the cost and blue stripped bars are used for the protection factor.

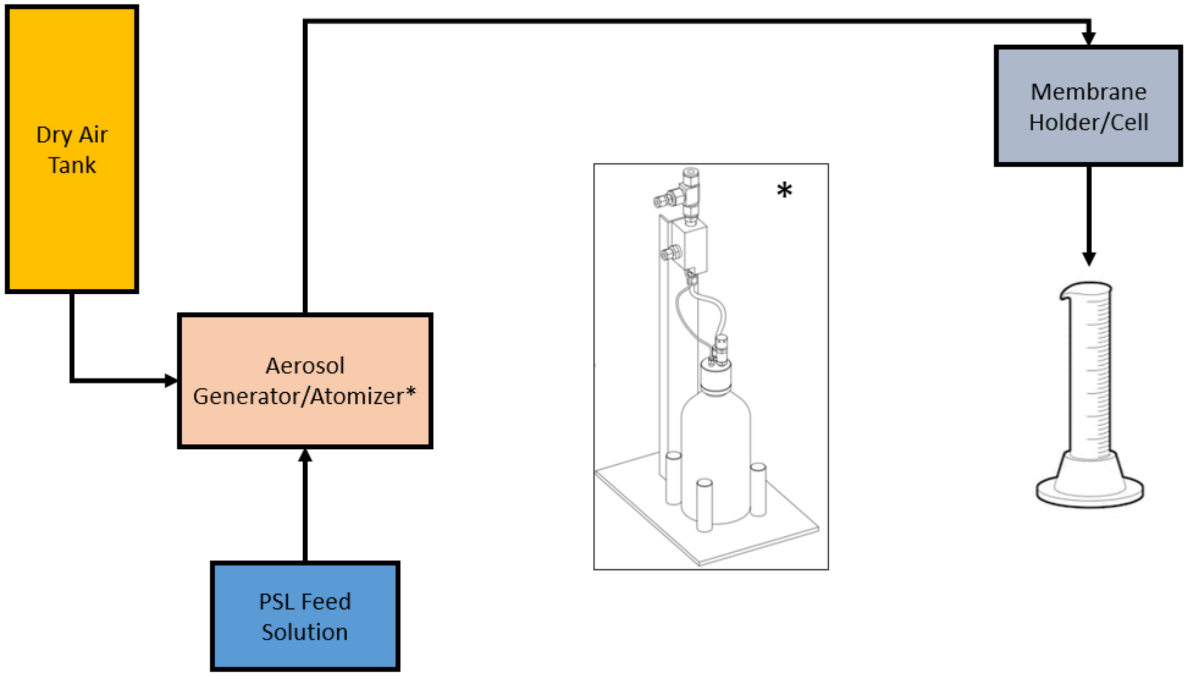

Supplementary Figure 15. A schematic of aerosol generation and filter testing module utilized in testing aerosolized particle filtration through membrane filters. Schematic of Aerosol Generator (TSI, Model 3076) provided from TSI manual.

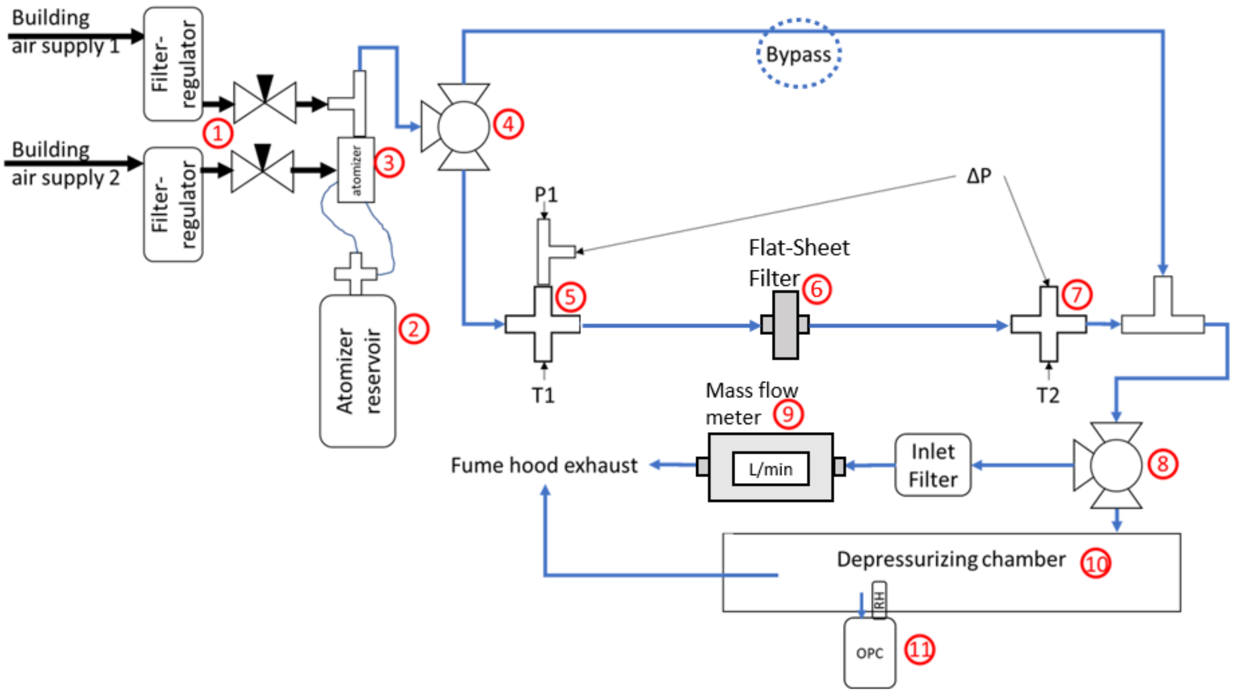

Supplementary Figure 16. Schematic of the aerosol filtration testing system with aerosol-phase measurements. The system consists of an atomizer feed solution used to generate aerosols, collision atomizer model 3076 (TSI), two dry filtered air streams from the building air supply which supply pressurized air to drive the atomizer and a drying stream to dry aerosols, a bypass ball valve to allow fast switching between filtered and unfiltered air streams for measurements, a switch ball valve to change the stream between optical particle counter and mass flow meter, and a depressurizing chamber for sampling by the optical particle counter. Tubing in the system is 3/8" I.D. teflon and fittings are brass or stainless steel. All tubing and fittings were purchased from McMaster Carr. (1) building air supply with filter-regulator and needle valves to control feed pressure, (2) atomizer feed reservoir bottle, (3) TSI model 3076 atomizer, (4) ball valve for switching to bypass for unfiltered aerosol measurement, (5) cross fitting with thermocouple and pressure gauges for upstream of filter, (6) filter being tested, (7) cross fitting with thermocouple and pressure gauges for downstream of filter, (8) ball valve for switching between mass flowmeter and optical particle counter analysis, (9) TSI model 4043 mass flow meter, and (10) depressurizing chamber to prevent overpressure to the optical particle counter (11). The effluent is vented to the fume hood as exhaust. All tubing and fittings were purchased from McMaster Carr. Aerosol system adapted from Baldridge, K. et al.[8]

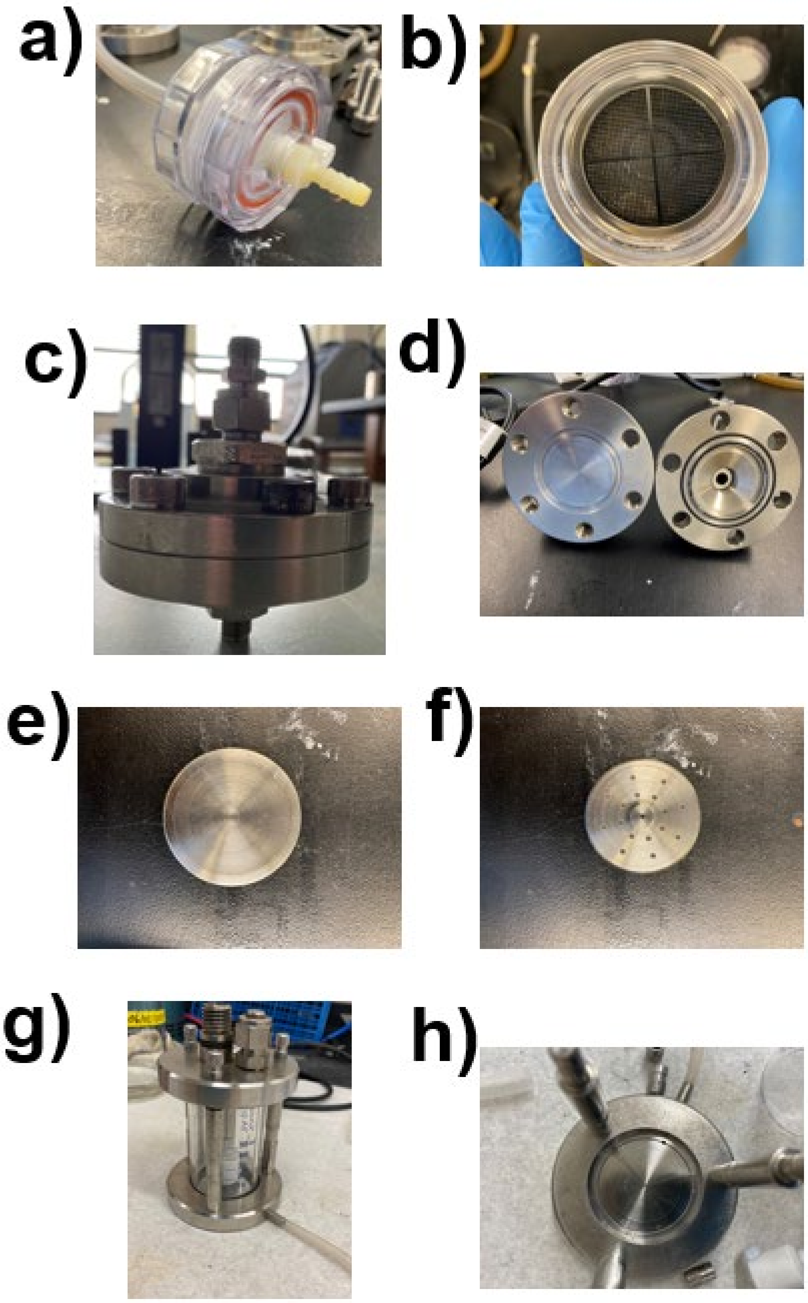

Supplementary Figure 17. Images of filtration cells utilized. (**a**) Polycarbonate air-filtration cell with (**b**) filter/membrane support mesh. (**c**) Inside and (**d**) outside of custom-made stainless-steel Honeywell filtration cell with (**e**) front and (**f**) back of mesh filter/membrane support. (**g**) Outside and (**h**) membrane holder area of Millipore dead-end stirred cell for water-filtration.

Supplementary Table 1. **Key membrane variables (porosity, thickness, mean pore diameter, and air permeability) of various commercial membranes**. Note Durapore membranes are hydrophilic. Flow rate measurements normalized at STP. PVDF400 thickness includes polyester backing support layer. Measurements taken in triplicate.

Membrane	Porosity	Thickness (μm)	Mean pore diameter (nm)	Air Permeability (LMH/bar)
PVDF400	0.46	165	45	$1.70\times10^6 \pm 6.80\times10^5$
Polycarbonate Isopore	0.138	25	200	$1.80\times10^6 \pm 3.71\times10^4$
Durapore1 (PVDF)	0.7	125	100	$6.49\times10^5 \pm 3.61\times10^3$
Durapore2 (PVDF)	0.7	125	220	$1.16\times10^6 \pm 7.38\times10^3$

Supplementary Table 2. Proposed membrane variables for calculated membrane air flux at 0.08 bar. Commercial N95 mask found to be 1.14E+06 LMH at 0.08 bar. Thickness stated does not include polyester support layer. Flow rate measurements normalized at STP. Flux measured in LMH.

PVDF Membrane	Porosity	PVDF Thickness (μm)	Mean pore diameter (nm)	Approximate air flux at 0.08 bar
Model Membrane 1	0.7	12.5-18.5	45	6.00E+05
Model Membrane 2	0.7	12.5-18.5	100	1.01E+06

Supplementary Table 3. Hydrodynamic diameter measurements (nm) of unfunctionalized polystyrene latex particles using Anton Paar Particle Analyzer (Litesizer 500). The concentration of the 100 nm and 200 nm particle solutions were 0.20 ppm and 0.15 ppm, respectively, at a pH of 7.3.

Stated Mean Diameter (nm)	Measured Hydrodynamic Diameter (nm)	Measured Hydrodynamic Diameter Range (nm)	Measured Hydrodynamic Diameter Peak (nm)
100	141.16	71.57 to 172.99	106.61
200	242.7	125.9 to 242.7	174.07

Supplementary Table 4. Experimentally-determined void fraction and estimated total pore volume of blank and PMAA-PVDF and PVDF400 membranes with an area 19.6 cm^2. PMAA-PVDF membranes had a weight gain of 1.2% - 2.9%.

Membrane	Void Fraction	Total Pore Volume (cm^3)
PVDF400	0.466	0.163
PMAA-PVDF	0.454	0.159

Supplementary Table 5. Maximum number of aerosolized 100-nm PSL particles captured by a blank and PMAA-functionalized PVDF400 membrane at two different particle packing structures.

Packing Structure	Membrane	Maximum Number Particles Captured
Hexagonal Lattice	PVDF400	1.25E+09
	PMAA-PVDF	1.22E+09
Face-Centered Cubic	PVDF400	1.53E+09
	PMAA-PVDF	1.50E+09

Supplementary Table 6. Statistical comparisons between paired samples to show changes in fluorescent intensity produced by Sypro Orange during SGP denaturation experimentation. Matched Pair T-Tests for means were performed using IBM SPSS-26 software.

Pairings	Mean Difference in RFU	95% Confidence Interval of the Difference	Significance of Difference (n=5)
Sypro Orange & Buffer on Membrane	65	(33,96)	P < .01
S-Protein (no membrane contact) & Adjusted S-Protein on Membrane	327	-292,362	P < .001

Supplementary References

1 Wan, H. *et al.* Pd/Fe nanoparticle integrated PMAA-PVDF membranes for chloro-organic remediation from synthetic and site groundwater. *Journal of Membrane Science* **594**, 117454, doi:https://doi.org/10.1016/j.memsci.2019.117454 (2020).

2 Penu, R. *et al.* Development of a nanocomposite system and its application in biosensors construction. *Central European Journal of Chemistry* **11**, 968-978, doi:10.2478/s11532-013-0222-7 (2013).

3 Ji, Y. *et al.* DFT-Calculated IR Spectrum Amide I, II, and III Band Contributions of N-Methylacetamide Fine Components. *ACS Omega* **5**, 8572-8578, doi:10.1021/acsomega.9b04421 (2020).

4 Sarma, R., Islam, M. S., Running, M. P. & Bhattacharyya, D. Multienzyme immobilized polymeric membrane reactor for transformation of lignin model compound. LID - 463 [pii] LID - 10.3390/polym10040463 [doi]. *Polymers* (2018).

5 Buising, K. L. *et al.* Use of portable air cleaners to reduce aerosol transmission on a hospital coronavirus disease 2019 (COVID-19) ward. *Infection Control & Hospital Epidemiology*, 1-6, doi:10.1017/ice.2021.284 (2021).

6 Duncan, S., Bodurtha, P. & Naqvi, S. The protective performance of reusable cloth face masks, disposable procedure masks, KN95 masks and N95 respirators: Filtration and total inward leakage. *PLoS One* **16**, e0258191-e0258191, doi:10.1371/journal.pone.0258191 (2021).

7 Mukerji, S. *et al.* Cost-effectiveness analysis of N95 respirators and medical masks to protect healthcare workers in China from respiratory infections. *BMC Infect Dis* **17**, 464-464, doi:10.1186/s12879-017-2564-9 (2017).

8 Baldridge, K. C. *et al.* Demonstration of Hollow Fiber Membrane-Based Enclosed Space Air Remediation for Capture of an Aerosolized Synthetic SARS-CoV-2 Mimic and Pseudovirus Particles. *ACS ES&T Engineering* **2**, 251-262, doi:10.1021/acsestengg.1c00369 (2022).
